# Exosomes derived from P2X7 receptor gene‐modified cells rescue inflammation‐compromised periodontal ligament stem cells from dysfunction

**DOI:** 10.1002/sctm.19-0418

**Published:** 2020-06-29

**Authors:** Xin‐Yue Xu, Bei‐Min Tian, Yu Xia, Yun‐Long Xia, Xuan Li, Huan Zhou, Yi‐Zhou Tan, Fa‐Ming Chen

**Affiliations:** ^1^ State Key Laboratory of Military Stomatology, National Clinical Research Center for Oral Diseases and Shaanxi Engineering Research Center for Dental Materials and Advanced Manufacture, Department of Periodontology School of Stomatology, Fourth Military Medical University Xi'an People's Republic of China; ^2^ Shaanxi Key Laboratory of Free Radical Biology and Medicine, The Ministry of Education Key Laboratory of Hazard Assessment and Control in Special Operational Environments Fourth Military Medical University Xi'an People's Republic of China; ^3^ Department of Cardiology Xijing Hospital, Fourth Military Medical University Xi'an People's Republic of China

**Keywords:** cell exosomes, gene therapy, inflammation, microenvironment, P2X7 receptor

## Abstract

Although cellular therapy has been proposed for inflammation‐related disorders such as periodontitis for decades, clinical application has been unsuccessful. One explanation for these disappointing results is that the functions of stem cells are substantially compromised when they are transplanted into an inflammatory in vivo milieu. Considering the previous finding that P2X7 receptor (P2X7R) gene modification is able to reverse inflammation‐mediated impairment of periodontal ligament stem cells (PDLSCs), we further hypothesized that cells subjected to P2X7R gene transduction also exert influences on other cells within an in vivo milieu via an exosome‐mediated paracrine mechanism. To define the paracrine ability of P2X7R gene‐modified cells, P2X7R gene‐modified stem cell‐derived conditional medium (CM‐Ad‐P2X7) and exosomes (Exs‐Ad‐P2X7) were used to incubate PDLSCs. In an inflammatory osteogenic microenvironment, inflammation‐mediated changes in PDLSCs were substantially reduced, as shown by quantitative real‐time PCR (qRT‐PCR) analysis, Western blot analysis, alkaline phosphatase (ALP) staining/activity assays, and Alizarin red staining. In addition, the Agilent miRNA microarray system combined with qRT‐PCR analysis revealed that miR‐3679‐5p, miR‐6515‐5p, and miR‐6747‐5p were highly expressed in Exs‐Ad‐P2X7. Further functional tests and luciferase reporter assays revealed that miR‐3679‐5p and miR‐6747‐5p bound directly to the GREM‐1 protein, while miR‐6515‐5p bound to the GREM‐1 protein indirectly; these effects combined to rescue inflammation‐compromised PDLSCs from dysfunction. Thus, in addition to maintaining their robust functionality under inflammatory conditions, P2X7R gene‐modified stem cells may exert positive influences on their neighbors via a paracrine mechanism, pointing to a novel strategy for modifying the harsh local microenvironment to accommodate stem cells and promote improved tissue regeneration.

## Graphical abstract

The potential mechanism involved in P2X7R‐mediated rescue of inflammation‐compromised periodontal ligament stem cells from dysfunction.

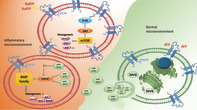

**Significance statement**

The authors' previous finding indicates that the P2X7 receptor (P2X7R) is a key molecule linked to inflammation‐related dysfunction of periodontal ligament stem cells (PDLSCs), and P2X7R‐gene‐modification can reverse or rescue the regenerative potential of PDLSCs under inflammatory conditions. Added to this, the present study for the first time proved that P2X7R‐gene modification cells could reverse inflammation‐mediated impairment of other coexisting cells via their secreted products. The data from this study suggest that exosomes derived from P2X7R‐gene‐modified cells could be used as an initiator to modify the local inflammatory microenvironment to accommodate stem cells, either outside transplanted or endogenously mobilized, toward an improved tissue regeneration.

## 1 INTRODUCTION

Periodontitis is a widespread disease characterized by inflammation‐induced connective tissue insult, intrabony pocket formation, damage to the tooth‐supporting apparatus, and eventual tooth loss (reviewed in Pihlstrom et al^1^). For over a decade, the idea of stem cell‐based periodontal therapy has brought hope for growing new tissues in periodontal pockets refractory to current regenerative paradigms, but the clinical translation of PDLSCs, the first cell source of choice for periodontal therapy, in patients suffering periodontitis has been disappointing to date (reviewed in Hu et al,^2^ Novello et al,^3^ and Nunez et al,^4^; eg, see Reference 5). One reason for the lack of success in clinical practice is that PDLSCs are functionally compromised when they are placed in an inflammatory intrabony periodontal defect.^4^, ^6^ Due to incomplete knowledge of the underlying mechanism,^7^, ^8^, ^9^ therapeutic approaches that protect PDLSCs from inflammation‐induced dysfunction (eg, reduced stemness and aberrant differentiation) following transplantation are still in their infancy.^10^, ^11^

In our previous study, we identified the role of P2X7 receptor (P2X7R) in inflammation‐compromised osteogenic differentiation of PDLSCs, and most importantly, we found that the cell impairment could be reversed by P2X7R gene modification.^8^ This finding suggests that the transplantation of P2X7R gene‐modified PDLSCs could lead to an improved therapeutic outcome. However, the large‐scale use of gene‐transferred cells in the clinic is associated with additional safety concerns. Further elucidation of the cellular and molecular events that occur when P2X7R gene‐modified PDLSCs encounter an inflammatory microenvironment is key to advance the P2X7R‐targeted strategy for cell‐based tissue regeneration.^12^

Growing evidence has demonstrated that exogenous transplanted cells may regulate and catalyze tissue regeneration via secretion of a spectrum of stimulatory factors as well as extracellular vesicles (EVs).^13^, ^14^, ^15^ In this regard, these cells, instead of or in addition to directly participating in the growth of new tissue, act as initiators to trigger and orchestrate tissue regeneration based on an endogenous repair mechanism, including but not limited to mobilizing/activating resident cells and modulating the regenerative microenvironment.^16^ In fact, the local inflammatory milieu not only impairs the therapeutic efficacy of transplanted cells but also negatively affects the regenerative potential of resident PDLSCs.^12^ Hence, it needs to be determined whether cells subjected to P2X7R gene transduction can exert positive influences on other coexisting resident cells within an in vivo milieu. If this is true, transplantation of a small number of cells subjected to P2X7R gene modification would be able to modulate the local hostile microenvironment and act as an initiator to trigger tissue repair in future periodontal regenerative medicine applications.

To define the secretory influence of P2X7R gene‐modified cells on other coexisting cells, the osteogenic potential of PDLSCs was investigated when they were placed in an inflammatory osteogenic microenvironment in the presence or absence of P2X7R gene‐modified stem cell‐derived conditional medium (CM‐Ad‐P2X7) and exosomes (Exs‐Ad‐P2X7). Furthermore, we explored the molecular changes in Exs‐Ad‐P2X7 and how the altered miRNAs were able to rescue inflammation‐compromised PDLSCs from dysfunction. Our data may provide new information for the development of microenvironment‐based strategies for tissue engineering and regeneration.

## 2 MATERIALS AND METHODS

### 2.1 Collection of cell conditioned media derived from various cultures

Human PDLSCs were isolated and modified to overexpress P2X7R by gene transfer methods (for additional details, see Appendix S1 in Supporting Information). Following culture for 24 hours in osteogenic culture medium (Cyagen Biosciences, Guangzhou, China), supernatants were collected from P2X7R gene‐transfected PDLSCs (Ad‐P2X7), PDLSCs transfected with blank adenoviral vectors (Ad‐control) and untransfected PDLSCs and designated as CM‐Ad‐P2X7, CM‐Ad‐control, and CM‐PDLSCs, respectively.

### 2.2 Effect of various conditioned media on PDLSCs under inflammatory conditions

#### 2.2.1 *Design of conditioned medium‐based cell cultures*

To test the effect of various conditioned media (CMs) on PDLSCs under inflammatory conditions, CM‐Ad‐P2X7, CM‐Ad‐control, and CM‐PDLSCs were mixed with osteogenic culture medium at a proportion of 1:1; fresh osteogenic culture medium without CMs was designed as the CM‐control.

#### 2.2.2 *Cell osteogenic differentiation in response to CM‐based incubation*

Following a 7‐day or 21‐day incubation, osteogenic differentiation of PDLSCs in CM‐based cultures (CM‐control, CM‐PDLSCs, CM‐Ad‐P2X7, and CM‐Ad‐control) was investigated in terms of osteogenic differentiation‐related gene/protein (BMP2, OCN, and Runx2) expression, alkaline phosphatase (ALP) staining/activity analysis, and Alizarin red staining (methods described in Appendix S1 in Supporting Information).

### 2.3 Isolation and identification of exosomes derived from P2X7R gene‐modified PDLSCs (Exs‐Ad‐P2X7)

#### 2.3.1 *Isolation of cell‐derived exosomes*

P2X7R gene‐modified PDLSCs (Ad‐ P2X7R) and cells transfected with blank adenoviral vectors (Ad‐control) were seeded in 100 mm culture dishes (Invitrogen, California) and cultured in serum‐free osteogenic inductive culture medium for 24 hours for exosome isolation. Exosomes were extracted by using the Total Exosome Isolation Kit for Cell Culture Media (Invitrogen, Lithuania) from supernatants according to the manufacturer's protocol. In brief, the obtained supernatants were centrifuged at 2000*g* for 30 minutes and then transferred to sterile tubes. Following the addition of the reagent to the supernatants and incubation overnight at 4°C, the mixture was centrifuged at 4°C and 10 000*g* for 1 hour. When the supernatant of the mixture was aspirated, exosomes attached to the tubes were suspended in PBS and normalized by cell number (the cells they were isolated from) to ensure that 100 μL of phosphate buffer saline (PBS) suspension contained exosomes isolated from 10^6^ cells.^17^ Exosomes derived from P2X7R gene‐modified PDLSCs were designated as Exs‐Ad‐P2X7R, while those derived from cells transfected with blank adenoviral vectors were designated as Exs‐Ad‐control.

#### 2.3.2 *Identification of Exs‐Ad‐P2X7*

The obtained exosomes (Exs‐Ad‐P2X7) were identified in terms of morphology (transmission electron microscopy [TEM]), surface markers (Western blot analysis), size (nanoparticle tracking analysis), and cell endocytosis experiments (Appendix S1 in Supporting Information).

### 2.4 Effects of Exs‐Ad‐P2X7 on PDLSCs under inflammatory conditions

#### 2.4.1 *Group design*

To test the effects of Exs‐Ad‐P2X7 on the osteogenic differentiation of PDLSCs under inflammatory conditions, exosomes were also isolated from PDLSCs transfected with blank adenoviral vectors (Exs‐Ad‐control) and untransfected PDLSCs (Exs‐PDLSCs); PBS was used as the blank control for Exs (Exs‐control). Finally, four groups (Exs‐control, Exs‐PDLSCs, Exs‐Ad‐P2X7, and Exs‐Ad‐control) were designed to investigate how the presence of Exs‐Ad‐P2X7 affects the osteogenic potential of PDLSCs.

#### 2.4.2 *Cell osteogenic differentiation in response to exosome‐based incubation*

Exs suspension (100 μL) was added to 2 × 10^5^ cells.^17^ Based on various culture conditions (Exs‐control, Exs‐PDLSCs, Exs‐Ad‐P2X7, and Exs‐Ad‐control), the osteogenic differentiation of PDLSCs under inflammatory conditions was tested following the determined investigation time in terms of osteogenic differentiation‐related gene/protein (BMP2, OCN, and Runx2) expression, ALP staining/activity analysis, and Alizarin red staining (Appendix S1 in Supporting Information).

### 2.5 Identification of differentially expressed miRNAs in Exs‐Ad‐P2X7

Total RNA from exosomes was extracted with TRIzol Reagent (Invitrogen) according to the instructions. RNA quantity and quality were measured by a Nano Drop ND‐1000 before miRNA sequencing. RNA labeling and array hybridization were performed according to the Agilent miRNA Microarray System with miRNA Complete Labeling and Hyb Kit protocol (Agilent Technology). The whole human miRNA microarray represents all known miRNAs in the human transcriptome. In brief, total miRNAs from Exs‐Ad‐P2X7 or Exs‐Ad‐control were labeled with Cyanine 3‐pCp under the action of T4 RNA ligase. The labeled cRNA was subjected to inspissation and desiccation and then dissolved in water. One microgram of each labeled cRNA was fragmented by adding 11 μL of 10X Blocking Agent and 2.2 μL of 25X Fragmentation Buffer and then heated at 60°C for 30 minutes; finally, 55 μL of 2X GE Hybridization buffer was added to dilute the labeled cRNA. Hybridization solution (100 μL) was dispensed into the gasket slide and assembled into the gene expression microarray slide. The slides were incubated for 17 hours at 65°C in an Agilent Hybridization Oven. The hybridized arrays were washed, fixed, and scanned using the Agilent Microarray Scanner (part number G2505C). Agilent Feature Extraction software (version 11.0.1.1) and the GeneSpring GX v12.1 software package (Agilent Technologies) were used to analyze and process the data. After quantile normalization of the raw data, miRNAs for which at least three out of six samples had flags in the detection system were chosen for further data analysis. Differentially expressed miRNAs with statistical significance between the two groups were identified through Volcano plot filtering and fold change filtering. Hierarchical clustering was performed using R scripts.

The active sequences of differentially expressed miRNAs are shown in Appendix S1 in Supporting Information. Then, quantitative real‐time PCR (qRT‐PCR) was used to validate the differentially expressed miRNAs as described in Appendix S1 in Supporting Information.

### 2.6 Effect of differentially expressed miRNAs in Exs‐Ad‐P2X7 on PDLSCs under inflammatory conditions

#### 2.6.1 *Transfection of PDLSCs with miRNA mimics related to differentially expressed miRNAs in Exs‐Ad‐P2X7*

To test the effects of differentially expressed miRNAs in Exs‐Ad‐P2X7 on PDLSCs under inflammatory conditions, miRNA mimics for differentially expressed miRNAs were purchased from Likeli Bioscience, Inc. (Likeli Bioscience, Inc., Beijing, China) and transfected into PDLSCs by Micropoly‐transfecter for cells (Micropoly, Nantong, China) according to the manufacturer's instructions. Cells transfected with the mimic‐negative control (Likeli, Beijing, China) were used as the negative control (miR‐mimic NC). Briefly, 1.5 μL of transfection reagent was mixed with 1.5 μL of 20 μM differentially expressed miRNA‐mimic or miR‐mimic NC and then incubated at room temperature for 10 minutes. The mixture was diluted in 300 μL of α‐MEM and added to 24‐well plates (cells at 40%‐50% confluence). The medium was replaced with osteogenic culture medium 12 hours after transfection. Total RNA was extracted at day 2 or day 7 for further analysis. All transfection experiments were repeated in triplicate.

#### 2.6.2 *Osteogenic differentiation of PDLSCs in response to miRNA‐mimic transfection*

The osteogenic differentiation of PDLSCs that were transfected with miR‐mimic NC or different miRNA mimics related to differentially expressed miRNAs between Exs‐Ad‐P2X7 and Exs‐Ad‐control were tested by means of qRT‐PCR (Appendix S1 in Supporting Information).

### 2.7 Determination of target genes for differentially expressed miRNAs in Exs‐Ad‐P2X7

#### 2.7.1 *Primary selection of target genes*

The potential genes targeted by differentially expressed miRNAs between Exs‐Ad‐P2X7 and Exs‐Ad‐control were assessed using the online software available from PicTar, TargetScan, and mirBase. The genes that were targeted by at least two differentially expressed miRNAs and closely related to osteoblast differentiation and osteogenesis were chosen for further validation using qRT‐PCR assays (for additional details, see Appendix S1 in Supporting Information).

#### 2.7.2 *Validation of the selected genes*

PDLSCs were seeded in six‐well plates at a concentration of 1 × 10^5^ cells/well, allowed to reach 80% confluence and then treated with Exs‐control, Exs‐PDLSCs, Exs‐Ad‐P2X7, and Exs‐Ad‐control for 7 days. The selected genes targeted by differentially expressed miRNAs in Exs‐Ad‐P2X7 was (were) validated by qRT‐PCR and Western blot assays. Then, PDLSCs were transfected with miR‐mimic NC or miRNA mimics of differentially expressed miRNAs, and the selected genes were tested by means of qRT‐PCR and Western blot assays (for additional details, see Appendix S1 in Supporting Information).

#### 2.7.3 *Luciferase reporter assay*

The final validation of the relationship between target genes and differentially expressed miRNAs was performed by using the luciferase reporter assay as described in Li et al.^18^ In brief, 293T cells were transfected with firefly luciferase transcript containing either a wild‐type or mutant form of the selected genes and then transfected with differentially expressed miRNA‐mimics; the luciferase reporter activity was assessed at 24 hours post‐transfection. A dual‐luciferase reporter assay system (Promega, Madison, Wisconsin) was used to measure the firefly and Renilla luciferase activities of the harvested cells. A luminometer (TD‐20/20; Turner Designs, Sunnyvale, California) was used to quantify luciferase activities and to calculate the relative ratios.

#### 2.7.4 *Elucidation of the functions of the target genes*

The functions of the selected genes were finally elucidated using the associated siRNA or plasmid. In brief, PDLSCs were seeded in six‐well plates at a concentration of 1 × 10^5^ cells/well; after the cells reached 80% confluence in six‐well dishes, siRNAs/plasmids for the target genes were transfected into PDLSCs using Micropoly‐transfecter for cells according to the manufacturer's instructions. siRNA NC/plasmid NC (Likeli, Beijing, China) was used as the negative control. The osteogenesis of different transfected PDLSCs was detected by BMP2, OCN, and Runx2 gene expression, as described in Appendix S1 in Supporting Information.

### 2.8 Statistical analysis

All results are presented as the mean and SD for at least n = 3; experiments for each cell line were performed independently and in triplicate. We used GraphPad Prism 7.03 software for statistical analysis. Student's *t* test was used to analyze two unpaired groups, and one‐way analysis of variance (ANOVA) followed by Tukey's post‐test was used for multiple group analysis. Statistical significance was established at *P* < .05.

## 3 RESULTS

### 3.1 Characterization of human PDLSCs

PDLSCs were isolated and characterized by their surface markers and multilineage potential, as reported previously.^8^ The isolated periodontal ligament (PDL) cells could multiply continuously (Figure S1B) and generate new colonies (Figure S1A). They were positive for the MSC markers CD90, CD105, CD146, and STRO‐1 and negative for the endothelial cell marker CD31 and the hematopoiesis‐related markers CD34 and CD45, as detected by flow cytometric analysis (Figure S1C). The isolated cells were able to differentiate under osteo‐inductive, adipo‐inductive, or chondrogenic inductive conditions, producing mineralized extracellular matrices positively stained with Alizarin red (Figure S1D), oil globules with abundant lipid‐rich vacuoles in the cytoplasm positively stained with Oil red O (Figure S1E), and acidic polysaccharide‐rich extracellular matrices positively stained with Alcian blue (Figure S1F). Accordingly, P2X7R‐gene‐modified PDLSCs were successfully acquired after the transduction of adenovirus vector at a concentration of 20 pfu/cell, as reported in our previous study.^8^ Western blot assays (Figure S2A) and immunofluorescence staining (Figure S2B) confirmed that P2X7R protein expression on PDLSCs was significantly increased after adenovirus vector transduction. In the flow cytometric analysis, Ad‐P2X7 transfected PDLSCs were positive for CD90, CD105, and CD146 while negative for CD31, CD34, and CD45 (Figure S2C). Compared to cells transfected with blank adenoviral vectors, P2X7R gene‐modified PDLSCs also possessed proliferative ability (Figure S2D) and multidifferential potential (Figure S2E‐G). These P2X7R gene‐modified PDLSCs were used for further studies.

### 3.2 Cell osteogenic differentiation in cultures containing CM derived from P2X7R gene‐modified stem cells

**FIGURE 1:**
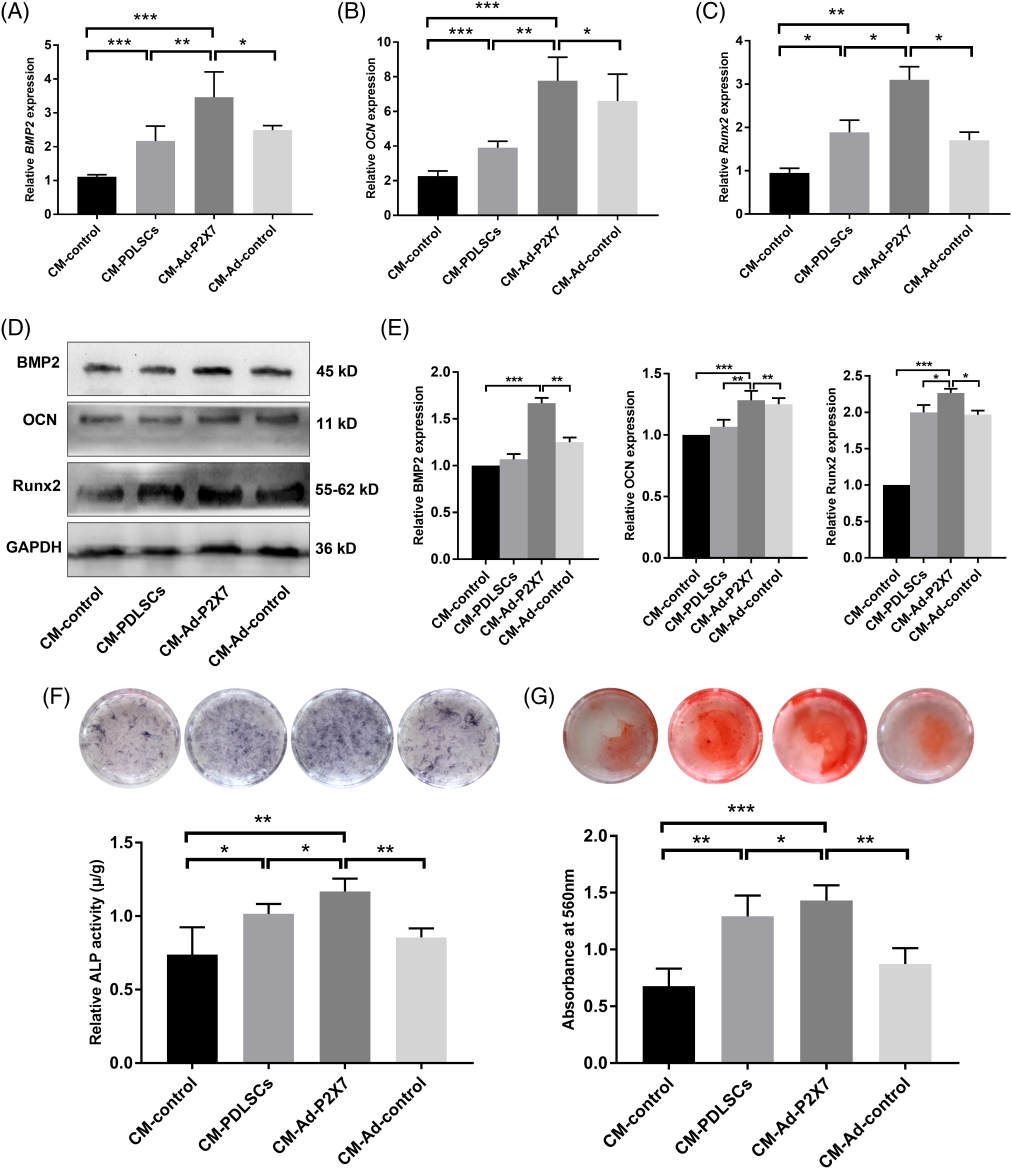
Incubation of PDLSCs in the presence of CM‐Ad‐P2X7 improved the osteogenic differentiation of PDLSCs in an inflammatory microenvironment. A, *BMP2*, B, *OCN*, and, C, *Runx2* gene expression in PDLSCs (qRT‐PCR assay) following a 7‐day incubation in cultures containing CM‐control, CM‐PDLSCs, CM‐Ad‐P2X7, and CM‐Ad‐control. D, BMP2, OCN, and Runx2 protein expression and, E, quantitative analysis in PDLSCs (Western blot analysis) following a 7‐day incubation in cultures containing CM‐control, CM‐PDLSCs, CM‐Ad‐P2X7, and CM‐Ad‐control. F, ALP staining and quantitative analysis of ALP activity in PDLSCs following a 7‐day incubation in cultures containing CM‐control, CM‐PDLSCs, CM‐Ad‐P2X7, and CM‐Ad‐control. G, Alizarin red staining and quantitative analysis of the mineralized nodes formed by PDLSCs following a 21‐day incubation in cultures containing CM‐control, CM‐PDLSCs, CM‐Ad‐P2X7, and CM‐Ad‐control. Data are presented as the mean ± SD for n = 4; *P < .05, **P < .01, and ***P < .001 indicate significant differences between the indicated columns. PDLSC, periodontal ligament stem cell

According to the study design, CM‐control, CM‐PDLSCs, CM‐Ad‐P2X7, and CM‐Ad‐control were used to culture PDLSCs in an inflammatory osteogenic microenvironment. The mRNA expression of osteogenic differentiation‐related genes (BMP2, OCN, and Runx2) was significantly increased in the CM‐Ad‐P2X7 group and slightly increased in the CM‐PDLSCs group and the CM‐Ad‐control group (*P* < .01, .05, or .001, Figure 1A; *P* < .01, .05, or .001, Figure 1B; *P* < .01 or .05, Figure 1C). The expression levels of *BMP2*, *OCN*, and *Runx2* were lowest in the CM‐control group (Figure 1A‐C). The Western blot analysis confirmed the results of qRT‐PCR, showing that BMP2, OCN, and Runx2 protein expression was significantly increased in the CM‐Ad‐P2X7 group but lower in the CM‐PDLSCs group and CM‐Ad‐control group (Figure 1D,E).

ALP or Alizarin red staining was used to analyze ALP activity and the mineralized nodules formed by different cultured PDLSCs in the presence of CM‐control, CM‐PDLCs, CM‐Ad‐P2X7, or CM‐Ad‐control. Both ALP staining/ALP activity and Alizarin red staining suggested that the CM‐Ad‐P2X7 group had better ALP activity and more mineralized nodule formation than the CM‐PDLSC group or the CM‐Ad‐control group (*P* < .01, .05, or .001, Figure 1F,G). The CM‐control group treated with fresh medium had the lowest ALP activity and least mineralized nodule formation.

### 3.3 Exosomes derived from P2X7R gene‐modified stem cells (Exs‐Ad‐P2X7)

**FIGURE 2:**
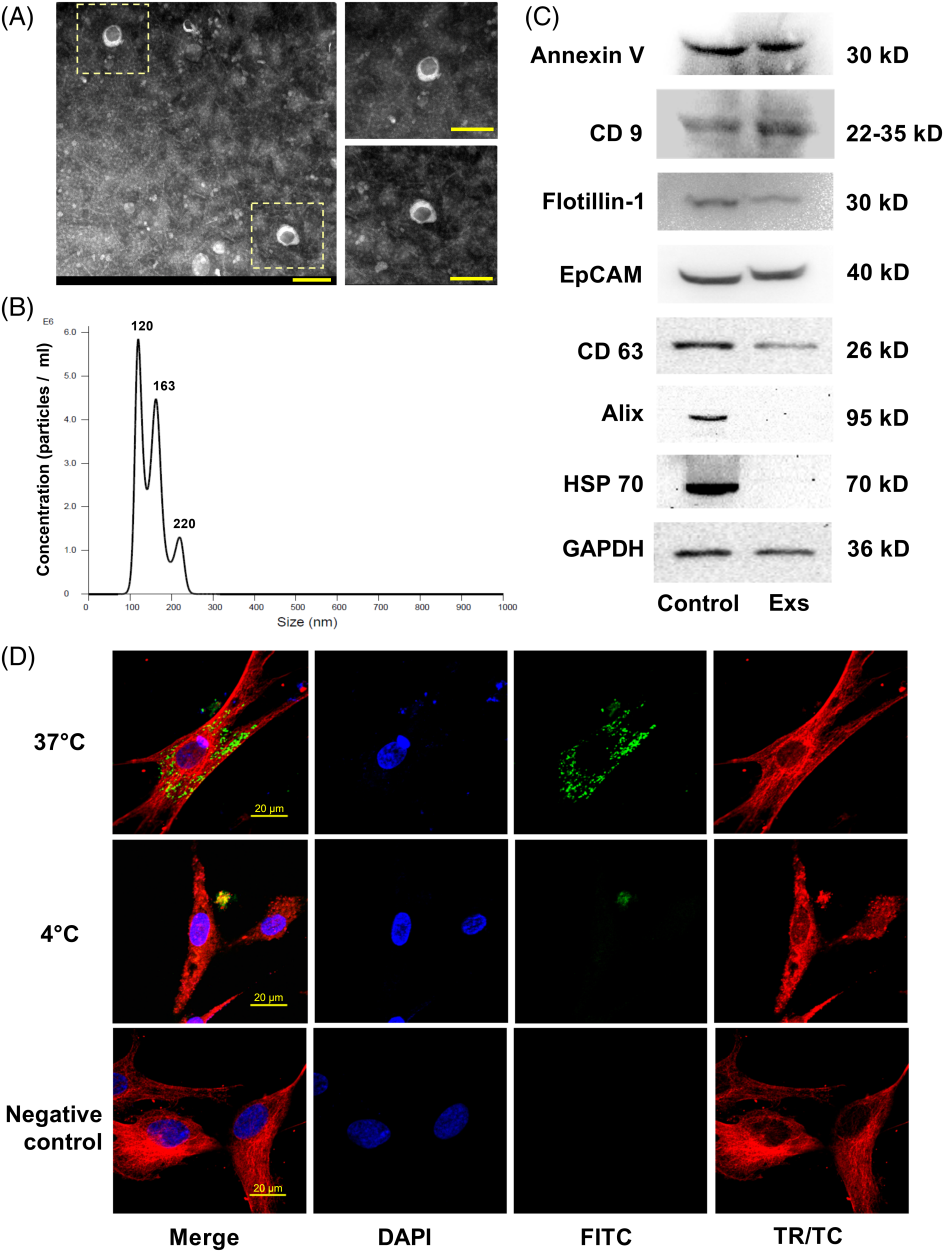
Characterization of exosomes derived from P2X7R gene‐modified stem cells. A, Representative TEM image of exosomes (scale bar = 200 nm). B, Size distribution profile of exosomes as determined by nanoparticle tracking analysis. C, Western blot analysis of cell lysates (control) and exosomes (Exs) showing the presence of the marker proteins Annexin V, CD 9, Flotillin‐1, EpCAM, and CD63 in both cell lysates and exosomes, while exosomes were negative for Alix and HSP70. D, Representative confocal image of fluorescently labeled exosomes (green) endocytosed by PDLSCs at 37°C or 4°C counterstained with tubulin (red) (scale bar = 20 μm). PDLSC, periodontal ligament stem cell; TEM, transmission electron microscopy

After 24 hours of culture in serum‐free osteogenic culture media, Ad‐P2X7‐transfected PDLSC culture supernatants were collected to isolate cell‐derived exosomes. When subjected to TEM observation, the obtained exosomes showed a cup‐ or sphere‐shaped morphology with a diameter in the range of 100 to 160 nm (Figure 2A). Nanosight analysis revealed that most isolated exosomes ranged in size from 120 to 163 nm (Figure 2B). Western blot analysis of exosomes and PDLSC lysates showed that the exosomes from Ad‐P2X7 transfected cells contained the exosomal markers Annexin V, CD9, Flotillin‐1, EpCAM, and CD63 (Figure 2C) but were negative for Alix and HSP70. The endocytosis of exosomes by PDLSCs is shown in Figure 2D. The isolated exosomes were labeled with PKH67 (green), and PDLSCs were incubated with the labeled exosomes at 37°C or 4°C for 4 hours. PDLSCs incubated without the labeled exosomes at 37°C were used as a negative control. After 4 hours, the cells were immunostained with anti‐tubulin antibody (red) and 4',6‐diamidino‐2‐phenylindole (DAPI; blue) to verify whether the exosomes could be endocytosed by PDLSCs. The images showed that in the control group (negative control), PDLSCs were negative for the green fluorescent signal, while the green fluorescent signals were significantly stronger in the exosome‐exposed group at 37°C; much lower green fluorescent signals were detected in the 4°C group.

### 3.4 Cell osteogenic differentiation in cultures containing Exs‐Ad‐P2X7

**FIGURE 3:**
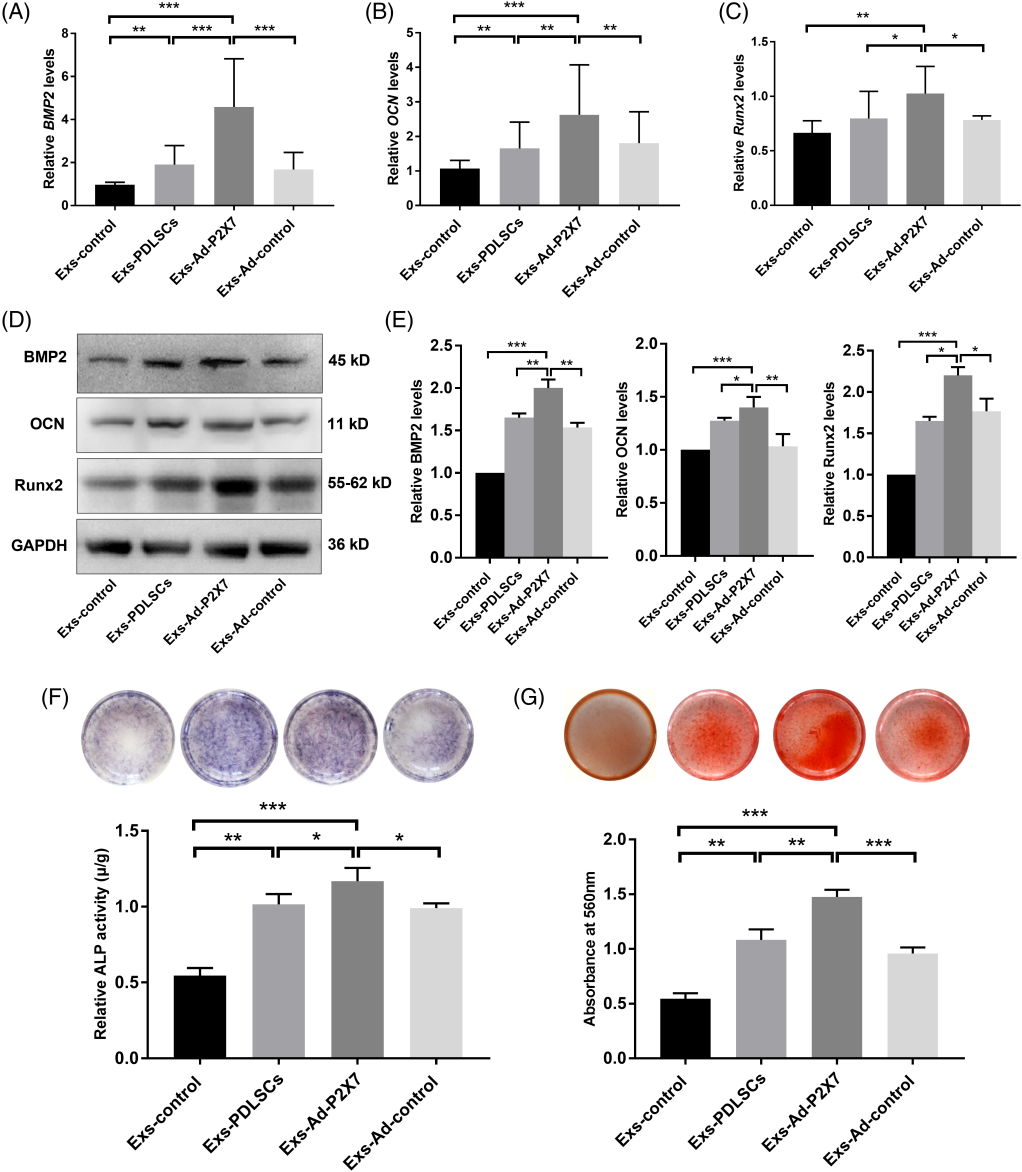
Incubation of PDLSCs in cultures containing Exs‐Ad‐P2X7 promoted osteogenic differentiation of PDLSCs in an inflammatory microenvironment. A, *BMP2*, B, *OCN*, and, C, *Runx2* gene expression in PDLSCs (qRT‐PCR assay) following a 7‐day incubation in cultures containing Exs‐control, Exs‐PDLSCs, Exs‐Ad‐P2X7, and Exs‐Ad‐control. D, BMP2, OCN, and Runx2 protein expression and, E, quantitative analysis in PDLSCs (Western blot analysis) following a 7‐day incubation in cultures containing Exs‐control, Exs‐PDLSCs, Exs‐Ad‐P2X7, and Exs‐Ad‐control. F, ALP staining and quantitative analysis of ALP activity in PDLSCs following a 7‐day incubation in cultures containing Exs‐control, Exs‐PDLSCs, Exs‐Ad‐P2X7, and Exs‐Ad‐control. G, Alizarin red staining and quantitative analysis of the mineralized nodes formed by PDLSCs following a 21‐day incubation in cultures containing Exs‐control, Exs‐PDLSCs, Exs‐Ad‐P2X7, and Exs‐Ad‐control. Data are presented as the mean ± SD for n = 4; *P < .05, **P < .01, and ***P < .001 indicate significant differences between the indicated columns. PDLSC, periodontal ligament stem cell

Ad‐P2X7‐ or Ad‐control‐transfected PDLSC‐derived exosomes (Exs‐Ad‐P2X7 or Exs‐Ad‐control) and untransfected PDLSC‐derived exosomes (Exs‐PDLSCs) were resuspended in PBS. Then, the exosome suspensions were normalized by cell number to ensure that 100 μL of suspension contained exosomes isolated from 1 million cells.^17^ PBS was used as the negative control group (Exs‐control). Then, the different exosome suspensions were added to inflammatory osteogenic culture medium for further incubation. After 7 days of incubation, *BMP2* expression was threefold higher in the Exs‐Ad‐P2X7 group than in the Exs‐control group (*P* < .001, Figure 3A). *Runx2* and *OCN* expression was also higher in the Exs‐Ad‐P2X7 group than in the other groups (Figure 3B,C). The Exs‐PDLSCs group and Exs‐Ad‐control group had similar *BMP2*, *Runx2*, and *OCN* expression levels, but all of these levels were higher than that of the Exs‐control group (Figure 3A‐C). The protein expression was consistent with mRNA expression (Figure 3D,E). The exosomes were also added to inflammatory osteogenic culture conditions, and PDLSCs were incubated for 7 or 21 days before the detection of ALP activity or Alizarin red staining. Both the ALP activity detection/staining and Alizarin red staining results suggested that the Exs‐Ad‐P2X7 group had better ALP activity and mineralized nodule formation than the Exs‐PDLSCs group or Exs‐Ad‐control group (*P* < .05, .01, or .001, Figure 3F,G). The ALP activity and mineralized nodule formation in the Exs‐control group were the lowest.

### 3.5 Identification of miR‐3679‐5p, miR‐6515‐5p, and miR‐6747‐5p as differentially expressed miRNAs in Exs‐Ad‐P2X7

**FIGURE 4:**
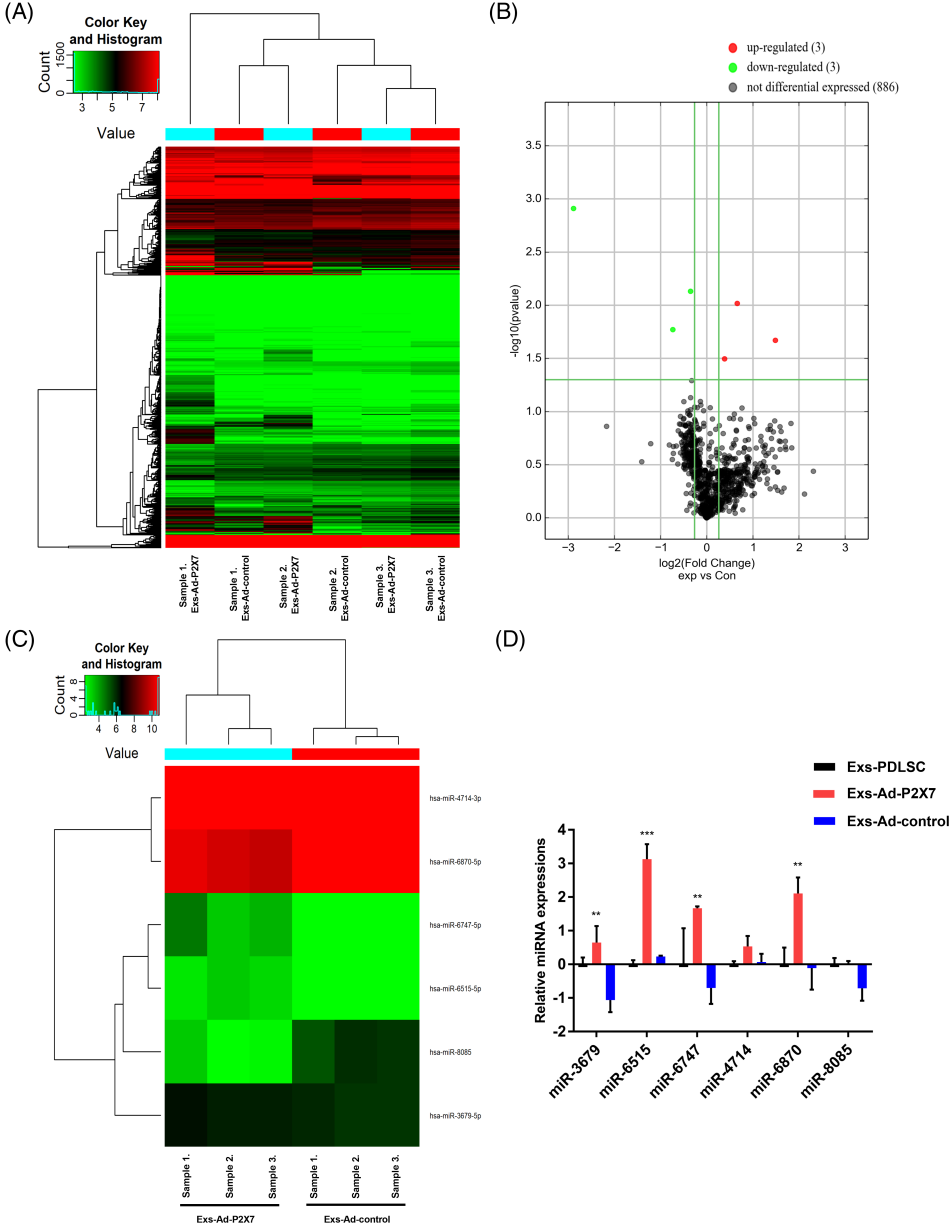
Differentially expressed miRNAs (miR‐3679‐5p, miR‐6515‐5p, and miR‐6747‐5p) in Exs‐Ad‐P2X7. A, Heat map of miRNA microarray expression data of exosomes derived from PDLSCs with or without P2X7R gene modification (Exs‐Ad‐P2X7 or Exs‐Ad‐control). B, Volcano plot (fold change > 1.2, *P* < .01); the red and green blocks indicate differentially increased or decreased miRNAs between Exs‐Ad‐P2X7 and Exs‐Ad‐control, and the gray blocks indicate miRNAs with no difference in expression. C, Heat map of differentially expressed miRNAs in Exs‐Ad‐P2X7 and Exs‐Ad‐control. D, Validation of the differentially expressed miRNAs in Exs‐Ad‐P2X7 and Exs‐Ad‐control by qRT‐PCR. Data are presented as the mean ± SD for n = 3; **P < .01 and ***P < .001 indicate significant differences between the indicated columns compared with the control group (PDLSC‐Exs). PDLSC, periodontal ligament stem cell

Exosomes from Ad‐P2X7R‐transfected PDLSCs carried cell‐specific miRNAs that mediated the functional effects of exosomes. We further investigated the miRNA profile of Exs‐Ad‐P2X7 and Exs‐Ad‐control from three different individuals. The data have been deposited in NCBI's Gene Expression Omnibus and are accessible through GEO Series accession number GSE140356 (https://www.ncbi.nlm.nih.gov/geo/query/acc.cgi?acc=GSE140356). Global miRNA profiling of Exs‐Ad‐P2X7 and Exs‐Ad‐control from three individuals demonstrated that there were six miRNAs with differential expression (Figure 4A‐C). Further qRT‐PCR analysis revealed that miR‐3679‐5p, miR‐6515‐5p, and miR‐6747‐5p were significantly increased in Exs‐Ad‐P2X7 compared with Exs‐Ad‐control (Figure 4D), in accordance with the sequencing results. The other three miRNAs were not consistent with the sequencing results. Thus, we used miR‐3679‐5p, miR‐6515‐5p, and miR‐6747‐5p as our study genes in subsequent experiments.

### 3.6 Differentially expressed miRNAs in Exs‐Ad‐P2X7 enhanced the osteogenic differentiation of PDLSCs in an inflammatory microenvironment

To explore the function of miR‐3679‐5p, miR‐6515‐5p, and miR‐6747‐5p in PDLSCs under inflammatory culture conditions, miRNA gain‐of‐function studies were carried out in PDLSCs with miRNA mimics for miR‐3679, miR‐6515, and miR‐6747 under inflammatory culture conditions. The appropriate transfection concentration for miRNA mimics was investigated by qRT‐PCR analysis (Figure S3). Then, PDLSCs were transfected with miR‐3679‐mimic, miR‐6515‐mimic, and miR‐6747‐mimic (MOI = 50) to characterize the effects of differentially expressed miRNAs on the osteogenic differentiation of PDLSCs.

**FIGURE 5:**
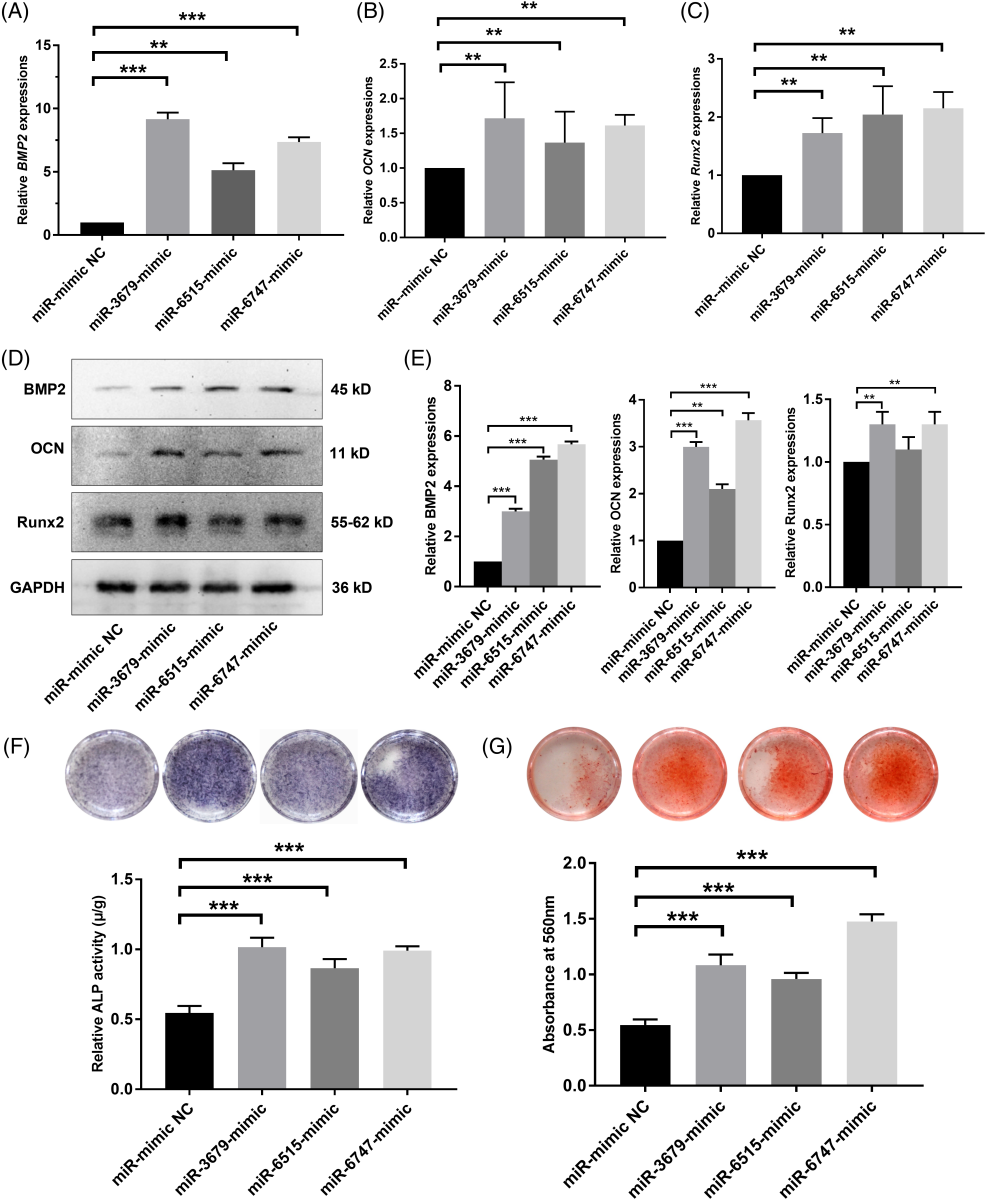
Transfection with miR‐3679‐mimic, miR‐6515‐mimic, or miR‐6747‐mimic promoted osteogenic differentiation of PDLSCs. A, *BMP2*, B, *OCN*, or, C, *Runx2* gene expression in PDLSCs (qRT‐PCR assay) following transfection with miR‐3679‐mimic, miR‐6515‐mimic, or miR‐6747‐mimic (the miRNA mimics that were related to the differentially expressed miRNAs identified in Exs‐Ad‐P2X7) and a 7‐day incubation in an inflammatory osteogenic microenvironment. D, BMP2, OCN, or Runx2 protein expression and, E, quantitative analysis in PDLSCs (Western blot analysis) following transfection with miR‐3679‐mimic, miR‐6515‐mimic, or miR‐6747‐mimic and a 7‐day incubation in an inflammatory osteogenic microenvironment. F, ALP staining and quantitative analysis of ALP activity in PDLSCs following transfection with miR‐mimic NC, miR‐3679‐mimic, miR‐6515‐mimic, or miR‐6747‐mimic and a 7‐day incubation in an inflammatory osteogenic microenvironment. G, Alizarin red staining and quantitative analysis of the mineralized nodes formed by PDLSCs following transfection with miR‐3679‐mimic, miR‐6515‐mimic, or miR‐6747‐mimic and a 21‐day incubation in an inflammatory osteogenic microenvironment. Data are presented as the mean ± SD for n = 4; *P < .05, **P < .01, and ***P < .001 indicate significant differences between the indicated groups and the control group (miR‐mimic NC). PDLSC, periodontal ligament stem cell

Further qRT‐PCR analysis suggested that the transduction of miR‐3679‐mimic, miR‐6515‐mimic, and miR‐6747‐mimic could significantly enhance the expression of *BMP2*, *OCN*, and *Runx2* compared with that of the control group (miR‐mimic NC) (Figure 5A‐C). *BMP2* expression in the miR‐3679‐mimic, miR‐6515‐mimic, and miR‐6747‐mimic groups increased 10‐fold, fivefold, and sevenfold, respectively. *OCN* and *Runx2* all increased over 1.2‐fold in each miR‐mimic group. However, the protein expression increase observed for Runx2 was not as obvious as the mRNA expression result, as the increase in Runx2 expression in all groups failed to reach statistical significance (Figure 5D,E). Both BMP2 and OCN protein expression increased over twofold in all miR‐mimic groups compared with the miR‐mimic NC group.

Both the ALP activity detection/staining and Alizarin red staining results suggested that differentially expressed miRNAs in Exs‐Ad‐P2X7 (miR‐3679‐5p, miR‐6515‐5p, and miR‐6747‐5p) could enhance ALP activity and mineralized nodule formation in PDLSCs under inflammatory culture conditions (*P* < .001; Figure 5F,G).

### 3.7 miR‐3679‐5p and miR‐6747‐5p binding to the GREM‐1 protein contributed to the cellular functions of Exs‐Ad‐P2X7

Prompted by these results, we then sought to investigate the molecular mechanism underlying Exs‐Ad‐P2X7‐enhanced osteogenic differentiation of PDLSCs in inflammatory microenvironments.

**FIGURE 6:**
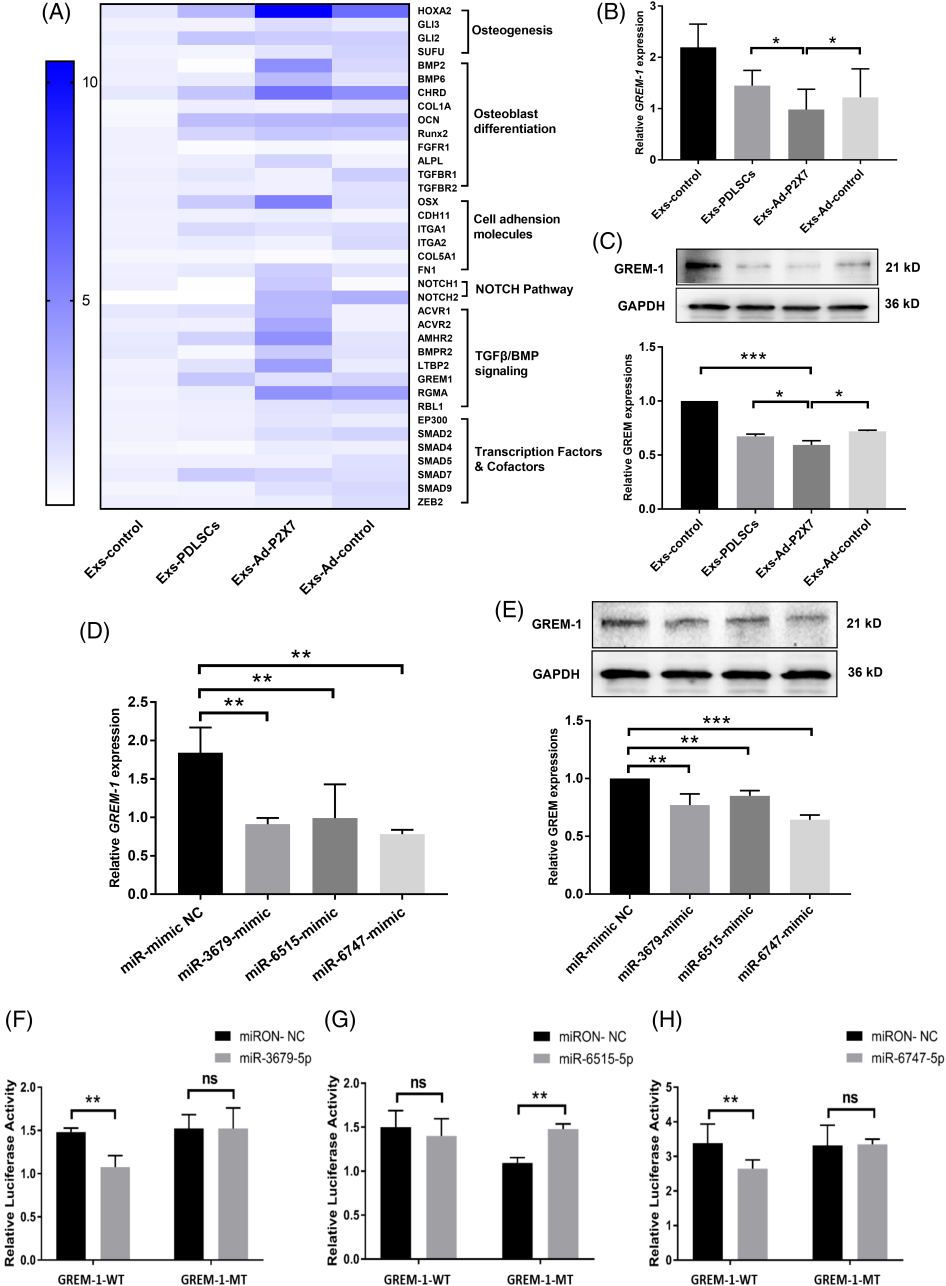
Determination of target genes related to the differentially expressed miRNAs (miR‐3679‐5p, miR‐6515‐5p, and miR‐6747‐5p) in Exs‐Ad‐P2X7. A, Osteogenic differentiation‐related signaling molecule expression of PDLSCs (qRT‐PCR assay) in inflammatory culture conditions in the presence of Exs‐control, Exs‐PDLSCs, Exs‐Ad‐P2X7, or Exs‐Ad‐control following a 7‐day incubation in an inflammatory osteogenic microenvironment. B, *GREM‐1* gene expression (qRT‐PCR assay) and, C, GREM‐1 protein expression (Western blot analysis) of PDLSCs under inflammatory culture conditions in the presence of Exs‐control, Exs‐PDLSCs, Exs‐Ad‐P2X7, or Exs‐Ad‐control following a 7‐day incubation in an inflammatory osteogenic microenvironment. D, *GREM‐1* gene expression (qRT‐PCR assay) and GREM‐1 protein expression (Western blot analysis) of PDLSCs following transfection with miR‐mimic NC, miR‐3679‐mimic, miR‐6515‐mimic, or miR‐6747‐mimic and a 7‐day incubation in an inflammatory osteogenic microenvironment. Relative luciferase activity of the GREM‐1 protein in, F, miR‐3679‐mimic‐transfected, G, miR‐6515‐mimic‐transfected, or, H, miR‐6747‐mimic‐transfected cells. Data are presented as the mean ± SD for n = 3; *P < .05, **P < .01, and ***P < .001 indicate significant differences between the indicated groups. PDLSC, periodontal ligament stem cell

Potential gene targets of miR‐3679‐5p, miR‐6515‐5p, and miR‐6747‐5p were assessed using the online software available from PicTar, TargetScan, and mirBase. More than 2000 genes were predicted to be potential targets of differentially expressed miRNAs. We focused on molecules that were closely related to osteogenic differentiation, and a survey was also performed using the PubMed website to find this miRNA function in other areas of study. Finally, 37 genes were selected and investigated by a qRT‐PCR assay (Figure 6A). The results suggested that some molecules, such as HOXA2, CHRD, OSX, LIFR, and EGFR, were significantly increased in Exs‐Ad‐P2X7‐treated PDLSCs in inflammatory osteogenic microenvironments, and among all the increased molecules, the transforming growth factor beta (TGF‐β)/bone morphogenetic protein (BMP) signaling family showed a similar increase in the Ad‐P2X7R‐Exs‐treated group. Thus, we hypothesized that the TGF‐β/BMP signaling pathway plays a central role in exosome‐enhanced osteogenic differentiation of PDLSCs in inflammatory microenvironments. GREM‐1, a BMP antagonist from the DAN family, plays a key role in regulating the TGF‐β/BMP signaling pathway. It can bind to BMPs and prevent BMPs from interacting with BMP receptors (provided by NCBI Reference Sequences; https://www.ncbi.nlm.nih.gov/gene/26585). Because GREM‐1 decreased significantly in Exs‐Ad‐P2X7‐treated PDLSCs (Figure 6B,C) and because transduction of miR‐3679‐mimic, miR‐6515‐mimic, and miR‐6747‐mimic into PDLSCs could also decrease GREM‐1 expression (Figure 6D,E), we hypothesized that GREM‐1 may be involved in miR‐3679‐, miR‐6515‐, and miR‐6747‐mediated changes in PDLSCs.

Furthermore, we used a luciferase reporter assay system to confirm the relationship between GREM‐1 and differentially expressed miR‐3679‐5p, miR‐6515‐5p, and miR‐6747‐5p. The results showed that the relative luciferase activity was reduced by 28% in the miR‐3679‐mimic group (*P* < .01; Figure 6F) and 22% in the miR‐6747‐mimic group compared with the miR‐3679‐mimic group (*P* < .01; Figure 6H), while transduction of the miR‐6515‐mimic did not change the relative luciferase activity compared with that of the NC‐mimic group (Figure 6G). This result suggests that GREM‐1 is the target gene for miR‐3679‐5p and miR‐6747‐5p but not for miR‐6515‐5p.

### 3.8 Inhibition and induction of GREM‐1 changed the osteogenic differentiation of PDLSCs under inflammatory conditions

**FIGURE 7:**
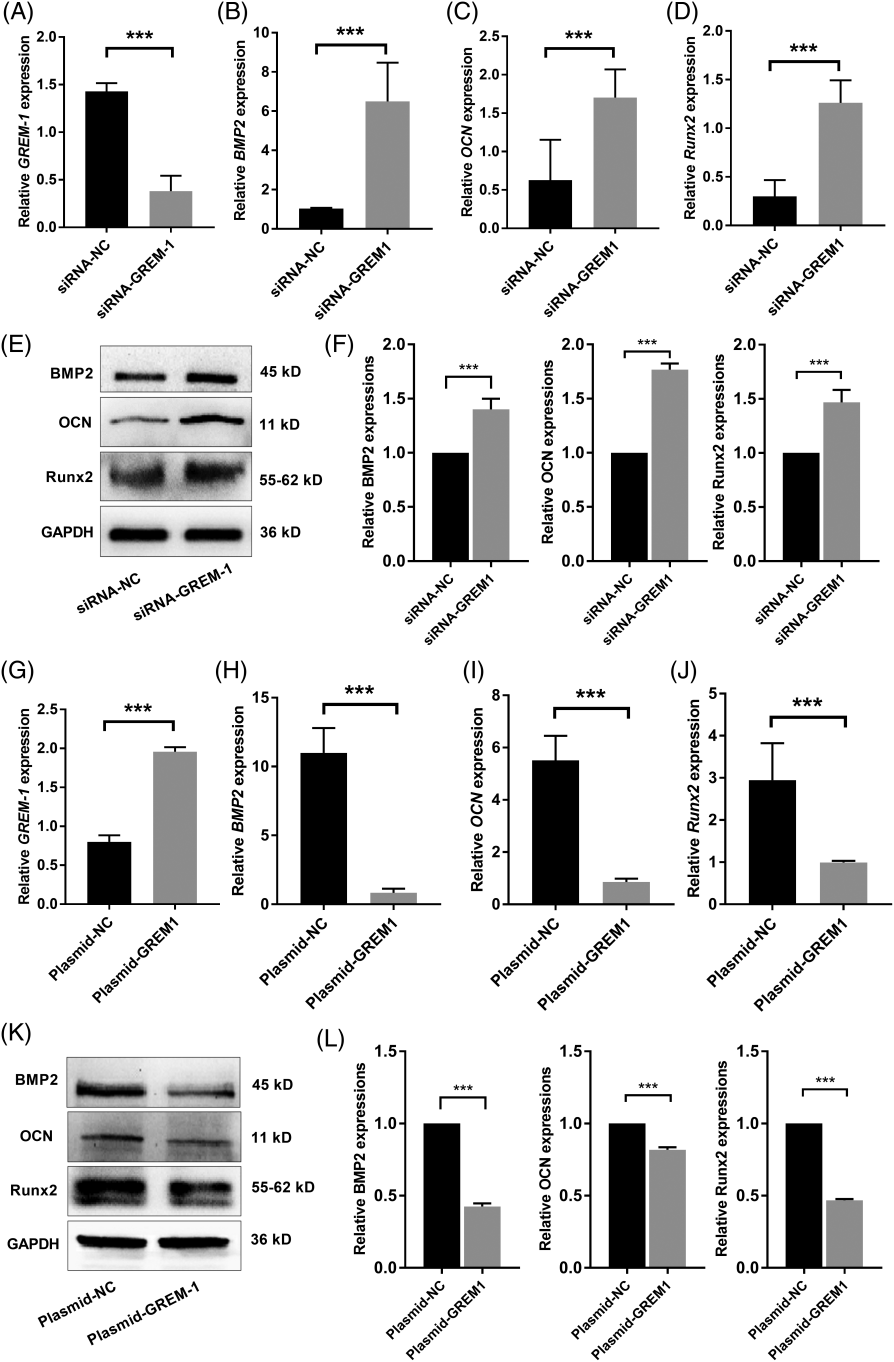
GREM‐1 gene modification by siRNA or plasmid changes the osteogenic differentiation of PDLSCs in an inflammatory microenvironment. A, *GREM‐1* gene expression (qRT‐PCR assay) in siRNA‐GREM‐1‐transfected PDLSCs following a 7‐day incubation in an inflammatory osteogenic microenvironment. B, *BMP2*, C, *OCN*, or, D, *Runx2* gene expression in siRNA‐GREM‐1‐transfected PDLSCs (qRT‐PCR assay) following a 7‐day incubation in an inflammatory osteogenic microenvironment. E, Protein expression and, F, quantitative analysis of BMP2, OCN, and Runx2 in siRNA‐GREM‐1‐transfected PDLSCs (Western blot analysis) following a 7‐day incubation in an inflammatory osteogenic microenvironment. G, *GREM‐1* gene expression (qRT‐PCR assay) in plasmid‐GREM‐1‐transfected PDLSCs following a 7‐day incubation in an inflammatory osteogenic microenvironment. H, *BMP2*, I, *OCN*, and, J, *Runx2* gene expression in plasmid‐GREM‐1‐transfected PDLSCs (qRT‐PCR assay) following a 7‐day incubation in an inflammatory osteogenic microenvironment. K, Protein expression and, L, quantitative analysis of BMP2, OCN, and Runx2 in Plasmid‐GREM‐1‐transfected PDLSCs (Western blot analysis) following a 7‐day incubation in an inflammatory osteogenic microenvironment. Data are presented as the mean ± SD for n = 3; *P < .05, **P < .01, and ***P < .001 indicate significant differences between the indicated columns. PDLSC, periodontal ligament stem cell

The function of GREM‐1 in osteogenic differentiation of PDLSCs in inflammatory microenvironments was further elucidated by GREM‐1 siRNA and plasmids. The expression of *GREM‐1* significantly decreased in the presence of siRNA‐GREM‐1, as detected by qRT‐PCR (Figure 7A). The mRNA expression of the osteogenic differentiation‐related genes *BMP2*, *OCN*, and *Runx2* were all increased in siRNA‐GREM‐1‐transfected PDLSCs (Figure 7B‐D). The protein expression of BMP2, OCN, and Runx2 was consistent with the mRNA expression results (Figure 7E,G). In contrast, the increase in GREM‐1 expression induced by Plasmid‐GREM‐1 in PDLSCs (Figure 7H) could decrease the expression of osteogenic differentiation‐related genes (Figure 7I‐M).

## 4 DISCUSSION

Multiple studies have proven that the inflammatory microenvironment in defective periodontal sites hinders stem cell‐based periodontal tissue regeneration.^4^, ^6^ Further elucidation of the underlying mechanism that occurs when stem cells encounter an inflammatory microenvironment will definitely benefit stem cell‐based regeneration therapy. The P2X7 receptor (P2X7R) belongs to the adrenergic receptor family^19^ and plays important physiological and pathological roles in vivo,^20^, ^21^, ^22^ including in periodontitis.^23^, ^24^ Our previous study proved that P2X7R gene modification could significantly enhance the osteogenesis of PDLSCs through the PI3K‐Akt‐mTOR signaling pathway.^8^

In fact, stem cells could improve the local microenvironment of the defect site not only by their own multiplication and differentiation but also by mobilizing the endogenous regeneration ability of resident cells though the molecules they secrete.^25^, ^26^ These transplanted stem cells can secrete a spectrum of stimulatory factors as well as EVs^13^, ^14^, ^15^ to enhance tissue regeneration. Coincidentally, the available literature supports the notion that P2X7R could enhance cell paracrine ability by enhancing cell membrane blebbing,^27^ cytokine release,^28^, ^29^ and exosome production in multiple cell types.^30^, ^31^, ^32^ To explore the potential modulatory effect of P2X7R gene‐modified PDLSCs on their neighbors when they coexist, conditioned medium from P2X7R gene‐modified stem cells (CM‐Ad‐P2X7) was collected and used to incubate PDLSCs in an inflammatory microenvironment. The results verified our hypothesis, showing that CM‐Ad‐P2X7 could significantly enhance the osteogenic differentiation of PDLSCs in an inflammatory microenvironment (Figure 1). In fact, previous studies have reported that stem cell‐derived CM could regulate the microenvironment of surrounding cells and improve cell function.^33‐36^ For example, CM from hypoxia‐preconditioned human periodontal ligament cells could suppress inflammation in experimental autoimmune encephalomyelitis, a murine model of multiple sclerosis.^33^ A similar study reported that CM from PDLSCs exert anti‐inflammatory effects in lipopolysaccharide (LPS)‐activated mouse motor neurons.^34^ Other studies reported that CM derived from mobilized dental pulp stem cells^35^ or apical tooth germ cells^36^ could promote cell proliferation, migration, and odontoblastic differentiation, thus facilitating pulp/dentin/cement regeneration.

CM contains multiple RNAs, secreted factors, growth factors, cytokines, and exosomes. In particular, exosomes, which range in size from 40 to 200 nm, are present in adequate amounts in multiple biological fluids, including cell culture medium,^37^ are relatively homogenous and have stable properties and low immunogenicity,^38^, ^39^ carry important messages for maintaining the internal microenvironment for regeneration.^40^, ^41^, ^42^, ^43^ Several reviews have summarized the use of exosomes as a potent therapeutic tool for regenerative medicine without inducing adverse immune reactions and pro‐inflammatory responses.^17^, ^38^, ^39^, ^40^ Furthermore, the function of exosomes can be markedly modified and improved by genetic manipulation, as reported by several studies,^44^, ^45^, ^46^ because they faithfully reflect the genomic characteristics of their parent cells and thus have functions similar to those of their parent cells.^37^, ^47^

In this study, we isolated exosomes (Exs‐Ad‐P2X7) from CM‐Ad‐P2X7 and explored their effects on stem cell behavior. The results showed that Exs‐Ad‐P2X7 had the best capacity to enhance the osteogenic differentiation of PDLSCs in an inflammatory microenvironment (Figure 3), which suggested that P2X7R gene‐modified stem cells have a stronger positive influence than unmodified stem cells on their coexisting neighbors. In fact, other studies also proved that genetic manipulation could enhance exosome function. In 2015, Kang et al^44^ revealed that exosomes derived from CXCR4‐overexpressing MSCs could better protect cardiomyocytes and increase angiogenesis in rat hearts after myocardial infarction. Yu's group proved that exosomes secreted by GATA‐4 gene‐modified MSCs contribute to cardiomyocyte survival and mitochondrial membrane potential preservation in a hypoxic environment.^45^ Another study reported that miR‐122‐transfected MSC‐derived exosomes could significantly increase the antitumor efficacy of sorafenib in hepatocarcinoma.^46^ A recent published review^48^ also mentioned that IL‐35 gene modification could change the content of MSC‐derived exosomes, which further induced the differentiation of Foxp3+ Treg cells. A recently published study^32^ reported that P2X7R could regulate bone marrow mesenchymal stem cells (BMMSC) and bone homeostasis through Tet1‐ and Tet2‐controlled P2X7R demethylation and exosome release.

The lipid bilayers of exosomes can effectively protect multiple RNAs from degradation. One previous study reported that most miRNAs in serum were present in exosomes.^49^ MicroRNA (miRNA) is a class of posttranscriptional regulator and one of the most important cargoes in exosomes.^50^ It is currently well recognized that miRNAs can bind to 60% of all genes and repress target gene expression,^51^, ^52^ regulating multiple physiological and pathological processes, including the osteogenic differentiation process.^53^, ^54^, ^55^ In this study, we sequenced the miRNA profiles of Exs‐Ad‐P2X7 and Exs‐Ad‐control from three different individuals and found that miR‐3679‐5p, miR‐6515‐5p, and miR‐6747‐5p increased significantly in Exs‐Ad‐P2X7 (Figure 4). Further study demonstrated that miR‐3679‐5p, miR‐6515‐5p, and miR‐6747‐5p played positive roles in the osteogenic differentiation of PDLSCs in the inflammatory microenvironment (Figure 5). A previous study reported that miR‐302/367 could maintain the pluripotency of human embryonic stem cells and regulate their differentiation.^56^ Liu et al^57^ reported that the expression of miR‐3679‐5p was significantly increased in plasma from coronary artery calcification patients in an independent clinical matched cohort. Another study reported that miR‐3679 and miR‐4274 were associated with bone mineral density in the osteoporotic phenotype.^58^ However, according to the author, the overexpression of miR‐3679 and miR‐4274 could contribute to the osteoporotic phenotype, which contradicted our results. The use of different cell sources and cultural environments, as well as different target genes of miRNAs, may cause inconsistent results. Until now, there were no more functional reports on miR‐6515 and miR‐6747, according to our PubMed search. Our study is the first to demonstrate that miR‐6515 and miR‐6747 derived from P2X7 are involved in the osteogenic differentiation of PDLSCs in an inflammatory microenvironment.

Based on current understanding, exosomes carrying miRNAs and transporting them between cells can significantly regulate cell growth and metabolism, most likely via posttranscriptional inhibition of gene expression.^59^ Hence, specific target genes of differentially expressed miRNAs in Exs‐Ad‐P2X7 were predicted by PicTar, TargetScan, and mirBase in the present study. Focusing on target genes of three differentially expressed miRNAs that were closely related to osteoblast differentiation and osteogenesis, we found that the TGF‐β/BMP signaling family was increased in the Ad‐P2X7R‐Exs‐treated group, suggesting that the TGF‐β/BMP signaling pathway plays a central role in exosome‐enhanced osteogenic differentiation of PDLSCs in inflammatory microenvironments. Because most miRNAs regulate cell function by posttranscriptional inhibition of gene expression, we then focused on investigating genes that were downregulated in the TGF‐β/BMP signaling family. We finally identified that GREM‐1 was most likely a common target gene of miRNA‐3679, miR‐6515, and miR‐6747. GREM‐1 is a BMP antagonist belonging to the DAN family. GREM‐1 binds to BMP2, BMP4, and BMP7 and inhibits BMP signaling.^60^ Further validation assays using a luciferase reporter assay confirmed that mechanistically, GREM‐1 was the direct target gene of the differentially expressed miRNAs miR‐3679‐5p and miR‐6747‐5p (Figure 6). A functional test of GREM‐1 suggested that there was an indirect connection between GREM‐1 and miR‐6515. Inhibition or induction of GREM‐1 changed the osteogenic differentiation of PDLSCs under inflammatory conditions (Figure 7). Similar results have been reported by Ghuman, showing that GREM‐1 can also limit coronal alveolar bone regenerative potential during oral and periodontal surgery by inhibiting BMP‐induced bone formation.^61^ Other studies have shown that GREM‐1 plays an important regularly role in organogenesis of the limbs and kidneys,^62^ and mutations in GREM‐1 lead to abnormalities of the limbs, lungs, and kidneys in mice.^63^

Taken together, these results show that P2X7R plays an important role in regulating and catalyzing tissue regeneration under inflammatory culture conditions. Cells subjected to P2X7R‐gene modification can exert a positive influence on other coexisting resident cells though the exosomes (Exs‐Ad‐P2X7) they secrete. Differentially expressed miRNAs in exosomes and their target gene GREM‐1 are involved in this process, as summarized in Figure S4. The utilization of Exs‐Ad‐P2X7 in the regeneration process instead of direct transplantation of adenovirus‐transfected stem cells into defective sites not only improves the safety and effectiveness of stem cell therapy without evoking additional side effects, but also provides a feasible and promising method for the clinical treatment of periodontitis. The limitations of the present study should also be acknowledged because the direct target gene of miR‐6515‐5p has not been identified, and more in vivo experiments are needed to verify the positive influence of P2X7R on tissue regeneration. However, with stringent proof‐of‐concept strategies, it might be possible to translate P2X7R gene modification from rodents to human patients.

## 5 CONCLUSION

In this study, we found that in addition to reversing inflammation‐mediated impairment in cells subjected to gene transduction, the transduced cells also exert positive influences on other coexisting cells via an exosome‐mediated paracrine mechanism. Our data suggest that either transplantation of a small number of P2X7R‐gene‐modified cells or the use of their exosomes could serve as an initiator to modify the local inflammatrory microenvironment to accommodate stem cells, either exogenously transplanted or endogenously mobilized, and lead to improved tissue regeneration.

## CONFLICT OF INTEREST

The authors declared no potential conflicts of interest.

## AUTHOR CONTRIBUTIONS

X.‐Y.X., B.‐M.T., Y.X.: conception/design, structure and figure design, collection and/or assembly of data, data analysis and interpretation, manuscript writing and revision, final approval of manuscript; F.‐M.C.: conception/design, structure and figure design, data analysis and interpretation, manuscript revision, final approval of manuscript; Y.‐L.X., X.L., H.Z., Y.‐Z.T.: data collection, data analysis and interpretation, manuscript writing, final approval of manuscript.

## Supporting information


**Appendix** **S1:** Supporting Information

**Supplemental Fig. 1** **Characterization of PDLSCs. (A)** Colony formation ability of PDLSCs: macroscopic view of colonies (left) and a single colony observed by microscopy (right, scale bar = 500 μm). **(B)** Proliferative activity of isolated PDLSCs assessed by a CCK‐8 assay. **(C)** Surface markers of PDLSCs assessed by flow cytometric analysis. **(D‐F)** Multilineage differentiation potential of PDLSCs demonstrated by Alizarin red staining (D), Oil red O staining (E) and Alcian blue staining (F) (scale bar = 500 μm).

**Supplemental Fig. 2** **Characterization of P2X7R gene‐modified PDLSCs (Ad‐P2X7R) using cells transfected with blank adenoviral vectors as the control (Ad‐control). (A)** P2X7R protein expression in PDLSCs transfected with Ad‐P2X7. **(B)** Representative confocal micrograph of gene‐modified PDLSCs carrying the green fluorescent protein (GFP) gene and P2X7R (immunofluorescence staining with a P2X7R antibody, red) (scale bar = 20 μm). **(C)** Surface markers of PDLSCs transfected with Ad‐P2X7 determined by flow cytometric analysis. **(D)** Proliferative activity of PDLSCs transfected with Ad‐P2X7 demonstrated by a CCK‐8 assay. **(E‐G)** Multilineage differentiation potential of PDLSCs transfected with Ad‐P2X7 demonstrated by **(E)** Alizarin red staining, **(F)** Oil red O staining and (G) Alcian blue staining (scale bar = 500 μm).

**Supplemental Fig. 3** Quantitative analysis of miRNA expression after PDLSCs were transfected with various concentrations (MOI = 20, 50 or 100) of miR‐3679‐mimic, miR‐6515‐mimic or miR‐6747‐mimic; untransfected cells (MOI = 0) were used as the control. Data are presented as the mean ± S.D. for *n* = 3; *P < 0.05, **P < 0.01 and ***P < 0.001 indicate significant differences between cells transfected with the indicated concentration of miRNA mimics (MOI = 20, 50 or 100) and untransfected cells (MOI = 0).

**Supplemental Fig. 4** **The potential mechanism involved in P2X7R‐mediated rescue of inflammation‐compromised PDLSCs from dysfunction.** Schematic illustration of how PI3K‐AKT‐mTOR signaling, exosomes, miR‐3679, miR‐6515 and miR‐6747 are involved in P2X7R‐mediated functional enhancement of PDLSCs living within an inflammatory microenvironment.

## Data Availability

The data supporting the findings of this study are available within the paper and its supplemental materials or from the authors upon request.
