# Supplementary material for: Exosomes derived from P2X7 receptor gene‐modified cells rescue inflammation‐compromised periodontal ligament stem cells from dysfunction
Source: Stem Cells Transl Med. 2020 Jun 29;9(11):1414–30. doi: 10.1002/sctm.19-0418 (PMC7581448; doi:10.1002/sctm.19-0418)
Supplement: Supplementary file 1 — Appendix S1: Supporting Information [file SCT3-9-1414-s001.docx]

**Appendix S1**

**Exosomes derived from P2X7 receptor gene-modified cells rescue inflammation-compromised periodontal ligament stem cells from dysfunction**

**Supplemental experimental procedures**

**1. Isolation, culture and characterization of PDLSCs**

***1.1. Cell isolation and culture***

Healthy human PDLSCs were obtained as reported in our previous study.^1^ In brief, PDLSCs were obtained from healthy extracted human third molars or orthodontic teeth from 10 individuals (6 males and 4 females), aged 18-40 years, without other systemic disease. Teeth were extracted at the Dental Clinic of the School of Stomatology, Fourth Military Medical University (FMMU). All individuals signed an informed consent form. The Ethics Committee of FMMU approved the study protocol. The extracted teeth were kept in sterile α-minimum essential medium (α-MEM, Invitrogen, USA) at 0°C for 2 h before transport to the lab. Then, the teeth were rinsed with sterile phosphate-buffered saline (PBS; Corning, New York, USA) before scraping the PDL tissue at the middle of the root surface. The blocks of PDL tissue were digested in 3 mg/mL collagenase type I (Sigma-Aldrich, St. Louis, USA) for 45 min with slight shaking. The samples were centrifuged, and the tissue at the bottom was collected and then cultured in 6-well plates (Invitrogen) in α-MEM containing 100 U/mL penicillin (Invitrogen), 100 μg/mL streptomycin (Invitrogen) and 10% fetal bovine serum (FBS; Sijiqing, Hangzhou, China). The medium was changed every 3 days after the primary cells migrated from the tissue blocks and achieved confluence using a limiting dilution technique to acquire purified PDLSCs. Purified PDLSCs at passages 2 to 5 (P2-P5) were used in the following experiments.

***1.2. Flow cytometry***

Flow cytometry was used to identify the surface markers of isolated PDLSCs. Briefly, 10^6^ cells/mL at P2 were incubated with different antibodies at 4°C for 1 h while protected from light according to the instructions. Specifically, antibodies against CD31 (#25-0319-42), CD34 (#17-0349-42), CD45 (#11-0452-82), CD90 (#11-0909-42), CD105 (#MA1-19594) and CD146 (#12-1469-42) were purchased from eBioscience^TM^ (eBioscience, CA, USA), and the antibody against Stro-1 (ab190282) was purchased from Abcam (Cambridge, MA, USA). Antibodies against mouse IgG were used as the negative staining isotype control. Then, excess antibodies were removed, and the PDLSCs were resuspended in 400 μL of PBS using a Beckman Coulter Epics XL cytometer (Beckman Coulter, Fullerton, CA, USA) for immunophenotype analysis.

***1.3. Colony formation assay***

The colony formation assay was performed with PDLSCs (P3); 1000 cells were plated into 100-mm culture dishes (Invitrogen), and the medium was changed every 3 days. After 2 weeks, the cells were fixed in 4% paraformaldehyde (Servicebio, Wuhan, China) and then stained with 0.1% crystal violet (Sigma-Aldrich) for 15 min. Then, we counted the colonies containing more than 50 cells under a microscope.

***1.4. Cell proliferation assay (CCK-8 assay)***

PDLSCs (P3) were plated into 96-well plates (Invitrogen) at a concentration of 2 × 10^4^ cells/well. Then, the PDLSCs were cultured in complete α-MEM for 24 h. Before the assay, Cell Counting Kit-8 solution (Dojindo Corporation, Tokyo, Japan) was prepared at a 1:100 dilution. Then, the solution was added to the well and incubated for 1 h before the absorbance was measured at 450 nm with an Infinite M200 PRO microplate reader (TECAN, Männedorf, Switzerland). Measurements were repeated for the next 7 days.

***1.5. Cell differentiation assay***

After PDLSCs reached 80% conﬂuence in 6-well dishes, they were cultured in osteoinductive medium, adipoinductive medium or chondrogenic differentiation medium to assess their multilineage differentiation capacity. The osteoinductive medium, adipoinductive medium and chondrogenic differentiation medium were purchased from Cyagen Biosciences (Cyagen Biosciences, Guangzhou, China). After a 21-day induction period (for osteoinduction or adipoinduction) or a 28-day induction period (for chondrogenic induction), differentiated PDLSCs were washed with PBS, fixed with paraformaldehyde and stained with Alizarin red (Cyagen) or Oil red O (Cyagen) to visualize the mineralized nodes or lipid droplets. The nodes of the cartilage were embedded in polysaccharide agarose and sliced before staining with Alcian blue (Cyagen).

**2. Creation of an inflammatory microenvironment**

The inflammatory microenvironment was created with TNF-α and IL-1β (Novoprotein Scientific Inc.,Shanghai, China) according to our previous study.^1^ Briefly, medium supplemented with TNF-α (at a concentration of 10 ng/mL) and IL-1β (at a concentration of 5 ng/mL) was used to establish an *in vitro* inflammatory microenvironment. Cells cultivated in parallel in a normal microenvironment were used as the control.

**3. Overexpression of P2X7R in PDLSCs using the gene transfer method**

We used adenoviral vectors (Likeli Bioscience Inc., Beijing, China) containing P2X7R cDNA (Ad-P2X7) to enhance P2X7R expression on PDLSCs as reported in our previous study.^1^ The multiplicity of infection (MOI) was 20 pfu/cell, and cells transfected with adenoviral vectors without P2X7R loading (Ad-control) were used as the control group. PDLSCs were incubated with adenovirus for 12 h and then washed with PBS three times before treatment with fresh α-MEM culture medium containing 10% FBS. The following experiments were performed 24 h after adenoviral infection.

**4. Osteogenic differentiation assay of PDLSCs incubated under various culture conditions**

PDLSCs at a concentration of 1 × 10^5^ cells/well were seeded in 6-well plates (Invitrogen) with α-MEM containing 10% FBS, 100 U/mL penicillin and 100 μg/mL streptomycin (Cyagen Biosciences, Guangzhou, China). After the PDLSCs reached 80% conﬂuence in 6-well dishes, they were subjected to the designated culture conditions. Then, osteogenic differentiation-related gene/protein (BMP2, OCN and Runx2) expression, ALP staining/activity and Alizarin red staining analyses were performed.

***4.1. qRT-PCR assays of BMP2, OCN and Runx2 mRNA expression***

qRT-PCR was used to investigate *BMP2, OCN* and *Runx2* mRNA expression after 7 days of culture under inflammatory osteoinductive culture conditions. Total RNA was extracted from the cells using the TRIzol™ Reagent (Invitrogen) according to the manufacturer’s instructions. Then, qRT-PCR analysis was used to detect *BMP2, OCN* and *Runx2* gene expression. Gene-specific qRT-PCR primers are shown in Supplemental Table 1.

***4.2. Western blot analysis of BMP2, OCN and Runx2 protein expression***

The total protein from PDLSCs was measured using a Protein Assay BCA Kit (Wako, Osaka, Japan) according to the manufacturer's instructions. Then, Western blot analysis was used to detect BMP2, OCN and Runx2 protein expression. Antibodies against BMP2 (#100221) were purchased from R&D (Minneapolis, MN, USA), antibodies against OCN (ab133612) were purchased from Abcam, and antibodies against Runx2 (#12556) were purchased from CST (Danvers, MA, USA).

***4.3. ALP staining and activity analysis***

ALP activity in CM- and Exs-treated PDLSCs or miRNA-mimic-transfected PDLSCs in inflammatory microenvironments was analyzed by ALP staining and activity analysis. In brief, after 7 days of culture in different osteoinductive culture media, a BCIP/NBT ALP staining kit (Beyotime Institute of Biotechnology, China) was used to stain the cells; an ALP assay kit (Beyotime Institute of Biotechnology, China) was used to quantitatively analyze ALP activity according to the manufacturer's instructions. In brief, the supernatant of the PDLSCs was collected. Then, p-nitrophenol, samples and pNPP were added to a 96-well plate and incubated in the dark at 37°C for 10 min. A stop solution was added to the plate before the absorbance was determined at 405 nm.

***4.4. Alizarin red staining and quantitative analysis***

After incubation with different osteogenic inductive media for 21 days, mineralized nodes were visualized by Alizarin red staining according to the manufacturer’s instructions (Heart, Xi’an, China). The mineralized nodes were dissolved in 2% cetylpyridinium chloride for 15 min, and absorbance was measured by the OD value at 560 nm for statistical analysis.

**5. Identification of Exs-Ad-P2X7**

***5.1. Transmission electron microscopy (TEM)***

The morphology of the exosomes was analyzed by TEM. Purified exosomes were fixed in 2% paraformaldehyde, negatively stained with 2% aqueous uranyl acetate solution and then loaded onto Formvar–carbon-coated grids. The exosomes were observed under a transmission electron microscope (JEOL JEM-1230, Tokyo Japan) at 80 kV.^2,3^

***5.2. Western blot analysis of surface markers***

Surface markers of exosomes were analyzed by Western blot analysis according to a previous study.^4^ The quantity of exosomes was measured by the total protein concentration of exosomes using a Protein Assay BCA Kit (Wako, Osaka, Japan) according to the manufacturer's instructions. Then, Western blot analysis was used to detect the surface markers of the exosomes. An Exosomal Marker Antibody Sampler Kit (#74220) was purchased from CST, and the CD63 antibody (#D360973) was purchased from Sangon Biotechnology Company (Shanghai, China).

***5.3. Nanoparticle tracking analysis (NTA)***

Exosomes from 10^7^ cells were diluted in 1 mL of PBS for nanoparticle tracking analysis. The size of isolated exosomes was determined though Nanosight Tracking Analysis with ZetaView PMX 110 (Particle Metrix, Meerbusch, Germany) according to a previously reported protocol.^5^

***5.4. Endocytosis experiments***

A PKH67 Green Fluorescent Cell Linker Mini Kit (Sigma Aldrich, St. Louis, MO, USA) was used to label the exosomes according to a previously published study.^6^ The exosomes were prepared according to the manufacturer’s instructions, and excess PKH67 dye was removed using exosome spin columns (Thermo Scientific Inc.). PKH67 dye without exosomes was used as a negative control solution. Then, 1 × 10^5^ PDLSCs were seeded onto a Nunc Glass Bottom Dish (Thermo Scientific Inc.) for 24 h before the experiment. Next, 50 μL of PKH67-labeled exosomes or a control mixture (PKH67 dye without exosomes) was added to the medium and incubated at 4°C or 37°C for 12 h. The dishes were washed 3 times with PBS and fixed in 4% paraformaldehyde (PFA) solution. The cytoskeleton was labeled with a Tubulin antibody (α/β-Tubulin Antibody; #2148; CST), and the nuclei were counterstained with DAPI (#D1306, Invitrogen). The cells were observed under a fluorescence microscope (DMI6000 B; Leica, Wetzlar, Germany) for image acquisition.

**6. qRT-PCR**

Total RNA was extracted from cells using the TRIzol™ Reagent (Invitrogen) according to the manufacturer’s instructions. Total RNA from exosomes was also extracted using the TRIzol™ Reagent (Invitrogen) as previously reported.^7^ The absorbance ratios at 260/280 nm and 260/230 nm were measured to evaluate the RNA quality. Total RNA was reverse transcribed using the Revert Aid First Strand cDNA Synthesis Kit (Yeasen Biotech Company) for mRNA or the miRNA First Strand cDNA Synthesis kit (Stem-loop Method) (Sangon Biotech, Shanghai, China) for miRNA. qRT-PCR was performed on cDNA samples using SYBR Green Master Mix (Yeasen Biotech Company) and a CFX Connect™ Real-Time PCR Detection System (Bio-Rad, Hercules, CA, USA), as reported in our previous study.^1^ Ampliﬁcation was performed under the following conditions: denaturation at 95°C for 5 min and 40 cycles at 95°C for 10 s, 55°C for 20 s and 72°C for 20 s, with melt curve analysis from 65°C to 95°C in increments of 0.5°C. Gene-specific qRT-PCR primers for miRNAs or mRNAs are shown in Supplemental Tables 1 and 2. The mRNA and miRNA expression levels for each gene were normalized and calculated using the ΔΔCt method. The relative expression level of a specific RNA was determined as using the formula 2^–ΔΔCt^.

**7. Western blot analysis**

The protein expression of PDLSCs or exosomes was measured by Western blot analysis. Briefly, total cellular protein was extracted with lysis buffer (Heart, China, Xi’an), and the protein concentration was measured using a BCA Protein Assay Kit (Heart). Exosome proteins were separated by sodium dodecyl sulfate polyacrylamide gel electrophoresis; for comparison, the same proteins were examined in PDLSC lysates. The proteins were transferred onto PVDF membranes (Millipore, Billerica, MA, USA), blocked in 5% BSA at room temperature for 1 h, incubated overnight at 4°C with primary antibodies and further incubated for 1 h at room temperature with an anti-rabbit or anti-mouse secondary antibody (CST). The blots were developed using Western Chemiluminescent HRP Substrate (EMD Millipore; Massachusetts, USA). Quantification of the final images was performed using Quantity One software (Version 4.6.2).

**Appendix S1 References**

1 Xu XY, He XT, Wang J et al. Role of the P2X7 receptor in inflammation-mediated changes in the osteogenesis of periodontal ligament stem cells. Cell Death Dis 2019;10:20.

2 Cizmar P, Yuana Y. Detection and characterization of extracellular vesicles by transmission and cryo-transmission electron microscopy. Methods Mol Biol 2017;1660:221–232.

3 Barbera-Cremades M, Gomez AI, Baroja-Mazo A et al. P2X7 receptor induces tumor necrosis factor-alpha converting enzyme activation and release to boost TNF-alpha production. Front Immunol 2017;8:862.

4 Huang CC, Narayanan R, Alapati S et al. Exosomes as biomimetic tools for stem cell differentiation: Applications in dental pulp tissue regeneration. Biomaterials 2016;111:103–115.

5 Johnsen KB, Gudbergsson JM, Skov MN et al. Evaluation of electroporation-induced adverse effects on adipose-derived stem cell exosomes. Cytotechnology 2016;68:2125–2138.

6 Komaki M, Numata Y, Morioka C et al. Exosomes of human placenta-derived mesenchymal stem cells stimulate angiogenesis. Stem Cell Res Ther 2017;8:219.

7 Momen-Heravi F, Bala S, Bukong T et al. Exosome-mediated delivery of functionally active miRNA-155 inhibitor to macrophages. Nanomedicine 2014;10:1517–1527.

**Supplemental Tables**

**Supplemental Table 1.** Primer sequences of mRNAs used in this study.

| Gene name | Gene  ID | Forward primer | Reverse primer |
| --- | --- | --- | --- |
| *BMP2* | 650 | ACTACCAGAAACGAGTGGGAA | GCATCTGTTCTCGGAAAACCT |
| *OCN* | 632 | CCCAGGCGCTACCTGTATCAA | GGTCAGCCAACTCGTCACAGTC |
| *RUNX2* | 860 | CACTGGCGCTGCAACAAGA | CATTCCGGAGCTCAGCAGAATAA |
| *GREM1* | 26585 | GCAAGTATCTGAAGCGAGATTG | CATCATGGTGGTGAACTTCTTG |
| *GAPDH* | 2597 | AGCCGCATCTTCTTTTGCGTC | TCATATTTGGCAGGTTTTTCT |
| *HOXA2* | 3199 | GTGGAGCTCCCACAAGTAGAA | TGGTTTTCCTTGCACTGGGT |
| *GLI2* | 2736 | GTGGAGCTCCCACAAGTAGAA | GAGTTGTGAGGCAGGCACTT |
| *GLI3* | 2737 | ACCACCAACATGGCTATCGG | TGATTTCCGTTGGTTGCAGTC |
| *SUFU* | 51684 | TCCAGATCGTTGGTGTCTGC | ACTCTCTCTTGCAGGTGTGG |
| *BMP6* | 654 | TGTTGGACACCCGTGTAGTAT | AACCCACAGATTGCTAGTGGC |
| *CHRD* | 8646 | CTTCGGCGGGAAGGTCTATG | GTCCCTGTCGCCTCGC |
| *COL1A* | 1277 | GAGGGCCAAGACGAAGACATC | CAGATCACGTCATCGCACAAC |
| *FGFR1* | 2260 | GTCTGCTGACTCCAGTGCAT | CTCCCAGGGGTTTGCCTAAG |
| *ALPL* | 249 | ACTGGTACTCAGACAACGAGAT | ACGTCAATGTCCCTGATGTTATG |
| *TGFBR1* | 7046 | TTTGCATCATCTCTGTCAAACG | TTGTGGATGATGTACTGAGTGG |
| *TGFBR2* | 7048 | TTCTTCATGTGTTCCTGTAGCT | TGACTAGCAACAAGTCAGGATT |
| *OSX* | 4281 | AAGGCAGTTGGCAGTAGTGG | TGAATGGGCTTCTTCCTCAG |
| *CDH11* | 1009 | GGGTGGGGAAGAAGACACAG | AGCCCAGGTCTAGGCATGTA |
| *ITGA1* | 3672 | ACGCTGCTGCGTATCATTCA | CACCTCTCCCAACTGGACAC |
| *ITGA2* | 3673 | GTGGCTTTCCTGAGAACCGA | GATCAAGCCGAGGCTCATGT |
| *COL5A1* | 1289 | CTTGGCCCAAAGAAAACCCG | GTAGGTGACGTTCTGGTGGG |
| *FN1* | 2335 | ACAAGCATGTCTCTCTGCCA | TCAGGAAACTCCCAGGGTGA |
| *NOTCH1* | 4851 | GGACGTCAGACTTGGCTCAG | ACATCTTGGGACGCATCTGG |
| *NOTCH2* | 4853 | GGGGTCCCCATTGCCTTAAT | GTTGGAGAGGCACTCGTTGA |
| *ACVR1* | 90 | TGTAATGGTCTCTGCTGCCC | GGGACCCGTGCTCATGATA |
| *ACVR2* | 92 | GCCGCGGAACATGACGG | GATGCACTCCCGTGTCTCAG |
| *AMHR2* | 269 | GATTTGAGGCCTGACAGCAG | GCCAGGTGGATGGGATGTAG |
| *BMPR2* | 659 | AGATCCTGGGCCATCAAAGC | TGACTCACTCTGTATACTGCTGC |
| *LTBP2* | 4053 | GGACGCGGAGTGTGTGAATA | ATCTGCTTGGTGATCCGCTG |
| *RGMA* | 56963 | CCACTCTGCAGTTGTCTCCC | CAAGGTGGGAGGGGCTC |
| *RBL1* | 5933 | AGCCGGAAGCAGAGGTAATTT | GTGGCTCTTCAGAAGCCGTA |
| *EP300* | 2033 | GCAGTGTGCCAAACCAGATG | GGGTTTGCCGGGGTACAATA |
| *SMAD2* | 4087 | GCCGGGAGGTTCGATACAAG | AGTCTCTTCACAACTGGCGG |
| *SMAD4* | 4089 | GGGACCGGATTACCCAAGAC | GTTAAGGGCCCCAACGGTAA |
| *SMAD5* | 4090 | CGCGAAAAGGAAGCTGTTGA | ACAAGTGCCATATGCTTCTTTCA |
| *SMAD7* | 4092 | ATGTTCAGGACCAAACGATCT | GGATGGTGGTGACCTTTGG |
| *SMAD9* | 4093 | ACGCCACCTATCCTGACTCT | ACTGGTCGAAAGTCTGAGTGT |
| *ZEB2* | 9839 | GAAGACAGAGAGTGGCATGTAT | GTGTGTTCGTATTTATGTCGCA |

**Supplemental Table 2.** Primer sequences of miRNAs used in this study.

| Gene name | Active sequence | RT primer | Forward primer | Reverse primer |
| --- | --- | --- | --- | --- |
| hsa-miR-3679-5p | TCCCCTTCCCTGCC | GTCGTATCCAGTGCAGGGTCCGAGGTATTCGCACTGGATACGACGGCAGG | CGCGCGCGTCCCCTTC | AGTGCAGGGTCCGAGGTATT |
| hsa-miR-4714-3p | CAACTCTGACCACCTAG | GTCGTATCCAGTGCAGGGTCCGAGGTATTCGCACTGGATACGACCAACTC | GCGCCAACCTAGGTGGTCA | AGTGCAGGGTCCGAGGTATT |
| hsa-miR-6515-5p | GATGTCTTCCACACCC | GTCGTATCCAGTGCAGGGTCCGAGGTATTCGCACTGGATACGACGGGTGT | CGCGCGGATGTCTTCC | AGTGCAGGGTCCGAGGTATT |
| hsa-miR-6747-5p | TGTTCTGCCTCTTTCCA | GTCGTATCCAGTGCAGGGTCCGAGGTATTCGCACTGGATACGACTGGAAA | CGCGCGTGTTCTGCCTC | AGTGCAGGGTCCGAGGTATT |
| hsa-miR-6870-5p | TCAACCCCCATCTCC | GTCGTATCCAGTGCAGGGTCCGAGGTATTCGCACTGGATACGACTCAACC | GCGTGGGGGAGATGGG | AGTGCAGGGTCCGAGGTATT |
| hsa-miR-8085 | GCCTCACAGTCCTCT | GTCGTATCCAGTGCAGGGTCCGAGGTATTCGCACTGGATACGACGCCTCA | CGCGTGGGAGAGAGGACTG | AGTGCAGGGTCCGAGGTATT |
| U6 |  | GTCGTATCCAGTGCAGGGTCCGAGGTATTCGCACTGGATACGACAAAATA | AGAGAAGATTAGCATGGCCCCTG | ATCCAGTGCAGGGTCCGAGG |
