# Supplementary material for: Exosomes derived from P2X7 receptor gene‐modified cells rescue inflammation‐compromised periodontal ligament stem cells from dysfunction
Source: Stem Cells Transl Med. 2020 Jun 29;9(11):1414–30. doi: 10.1002/sctm.19-0418 (PMC7581448; doi:10.1002/sctm.19-0418)
Supplement: Supplementary file 2 — Supplemental Fig. 1 Characterization of PDLSCs. (A) Colony formation ability of PDLSCs: macroscopic view of colonies (left) and a single colony observed by microscopy (right, scale bar = 500 μm). (B) Proliferative activity of isolated PDLSCs assessed by a CCK‐8 assay. (C) Surface markers of PDLSCs assessed by flow cytometric analysis. (D‐F) Multilineage differentiation potential of PDLSCs demonstrated by Alizarin red staining (D), Oil red O staining (E) and Alcian blue staining (F) (scale bar = 500 μm). [file SCT3-9-1414-s002.docx]

**Supplementary Figure. 1.**


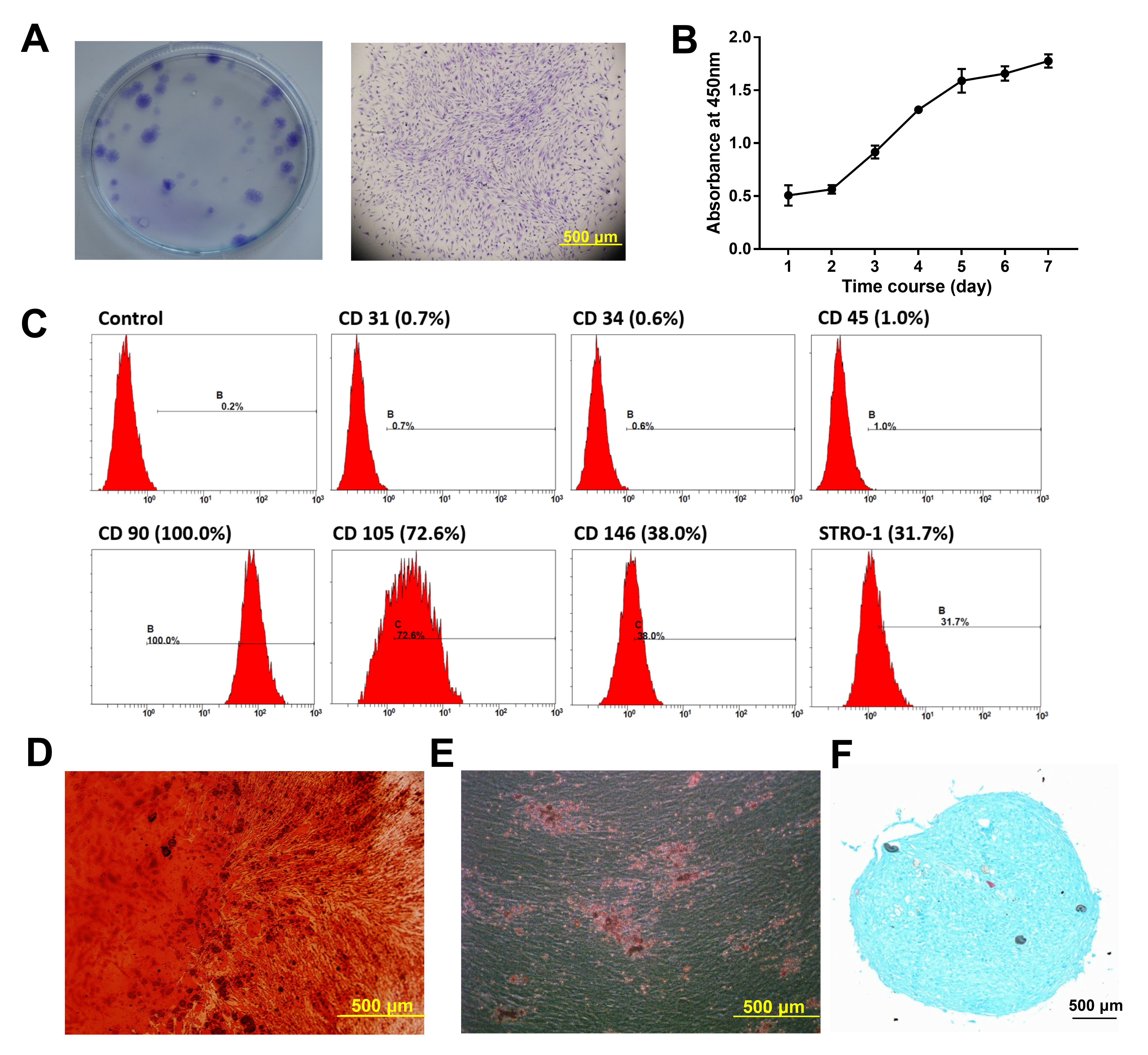


**Supplemental Fig. 1. Characterization of PDLSCs. (A)** Colony formation ability of PDLSCs: macroscopic view of colonies (left) and a single colony observed by microscopy (right, scale bar = 500 μm). **(B)** Proliferative activity of isolated PDLSCs assessed by a CCK-8 assay. **(C)** Surface markers of PDLSCs assessed by flow cytometric analysis. **(D-F)** Multilineage differentiation potential of PDLSCs demonstrated by Alizarin red staining (D), Oil red O staining (E) and Alcian blue staining (F) (scale bar = 500 μm).
