# Supplementary material for: Exosomes derived from P2X7 receptor gene‐modified cells rescue inflammation‐compromised periodontal ligament stem cells from dysfunction
Source: Stem Cells Transl Med. 2020 Jun 29;9(11):1414–30. doi: 10.1002/sctm.19-0418 (PMC7581448; doi:10.1002/sctm.19-0418)
Supplement: Supplementary file 3 — Supplemental Fig. 2 Characterization of P2X7R gene‐modified PDLSCs (Ad‐P2X7R) using cells transfected with blank adenoviral vectors as the control (Ad‐control). (A) P2X7R protein expression in PDLSCs transfected with Ad‐P2X7. (B) Representative confocal micrograph of gene‐modified PDLSCs carrying the green fluorescent protein (GFP) gene and P2X7R (immunofluorescence staining with a P2X7R antibody, red) (scale bar = 20 μm). (C) Surface markers of PDLSCs transfected with Ad‐P2X7 determined by flow cytometric analysis. (D) Proliferative activity of PDLSCs transfected with Ad‐P2X7 demonstrated by a CCK‐8 assay. (E‐G) Multilineage differentiation potential of PDLSCs transfected with Ad‐P2X7 demonstrated by (E) Alizarin red staining, (F) Oil red O staining and (G) Alcian blue staining (scale bar = 500 μm). [file SCT3-9-1414-s003.docx]

**Supplementary Figure. 2.**


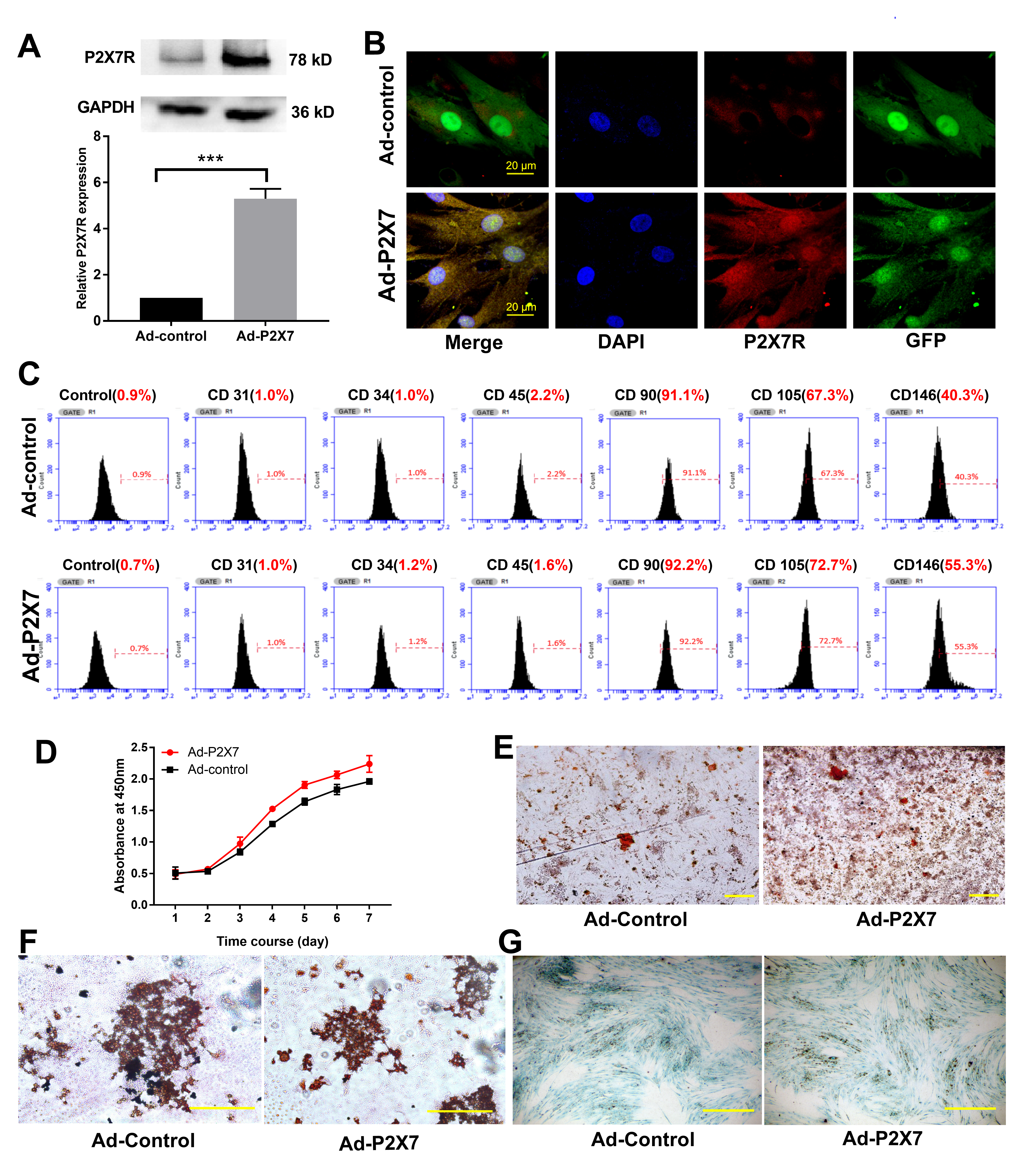


**Supplemental Fig. 2. Characterization of P2X7R gene-modified PDLSCs (Ad-P2X7R) using cells transfected with blank adenoviral vectors as the control (Ad-control). (A)** P2X7R protein expression in PDLSCs transfected with Ad-P2X7. **(B)** Representative confocal micrograph of gene-modified PDLSCs carrying the green fluorescent protein (GFP) gene and P2X7R (immunofluorescence staining with a P2X7R antibody, red) (scale bar = 20 μm). **(C)** Surface markers of PDLSCs transfected with Ad-P2X7 determined by flow cytometric analysis. **(D)** Proliferative activity of PDLSCs transfected with Ad-P2X7 demonstrated by a CCK-8 assay. **(E-G)** Multilineage differentiation potential of PDLSCs transfected with Ad-P2X7 demonstrated by **(E)** Alizarin red staining, **(F)** Oil red O staining and **(G)** Alcian blue staining (scale bar = 500 μm).
