# Supplementary material for: Exosomes derived from P2X7 receptor gene‐modified cells rescue inflammation‐compromised periodontal ligament stem cells from dysfunction
Source: Stem Cells Transl Med. 2020 Jun 29;9(11):1414–30. doi: 10.1002/sctm.19-0418 (PMC7581448; doi:10.1002/sctm.19-0418)
Supplement: Supplementary file 4 — Supplemental Fig. 3 Quantitative analysis of miRNA expression after PDLSCs were transfected with various concentrations (MOI = 20, 50 or 100) of miR‐3679‐mimic, miR‐6515‐mimic or miR‐6747‐mimic; untransfected cells (MOI = 0) were used as the control. Data are presented as the mean ± S.D. for n = 3; *P < 0.05, **P < 0.01 and ***P < 0.001 indicate significant differences between cells transfected with the indicated concentration of miRNA mimics (MOI = 20, 50 or 100) and untransfected cells (MOI = 0). [file SCT3-9-1414-s004.docx]

**Supplementary Figure. 3.**


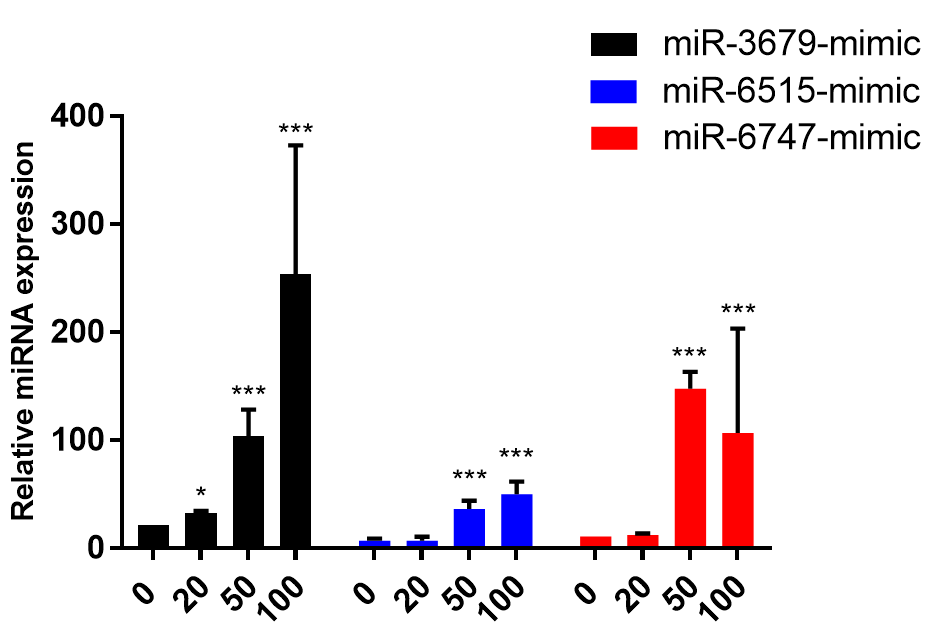


**Supplemental Fig. 3.** Quantitative analysis of miRNA expression after PDLSCs were transfected with various concentrations (MOI = 20, 50 or 100) of miR-3679-mimic, miR-6515-mimic or miR-6747-mimic; untransfected cells (MOI = 0) were used as the control. Data are presented as the mean ± S.D. for *n* = 3; **p* < 0.05, ***p* < 0.01 and ****p* < 0.001 indicate significant differences between cells transfected with the indicated concentration of miRNA mimics (MOI = 20, 50 or 100) and untransfected cells (MOI = 0).
