# Supplementary material for: Exosomes derived from P2X7 receptor gene‐modified cells rescue inflammation‐compromised periodontal ligament stem cells from dysfunction
Source: Stem Cells Transl Med. 2020 Jun 29;9(11):1414–30. doi: 10.1002/sctm.19-0418 (PMC7581448; doi:10.1002/sctm.19-0418)
Supplement: Supplementary file 5 — Supplemental Fig. 4 The potential mechanism involved in P2X7R‐mediated rescue of inflammation‐compromised PDLSCs from dysfunction. Schematic illustration of how PI3K‐AKT‐mTOR signaling, exosomes, miR‐3679, miR‐6515 and miR‐6747 are involved in P2X7R‐mediated functional enhancement of PDLSCs living within an inflammatory microenvironment. [file SCT3-9-1414-s005.docx]

**Supplementary Figure. 4.**


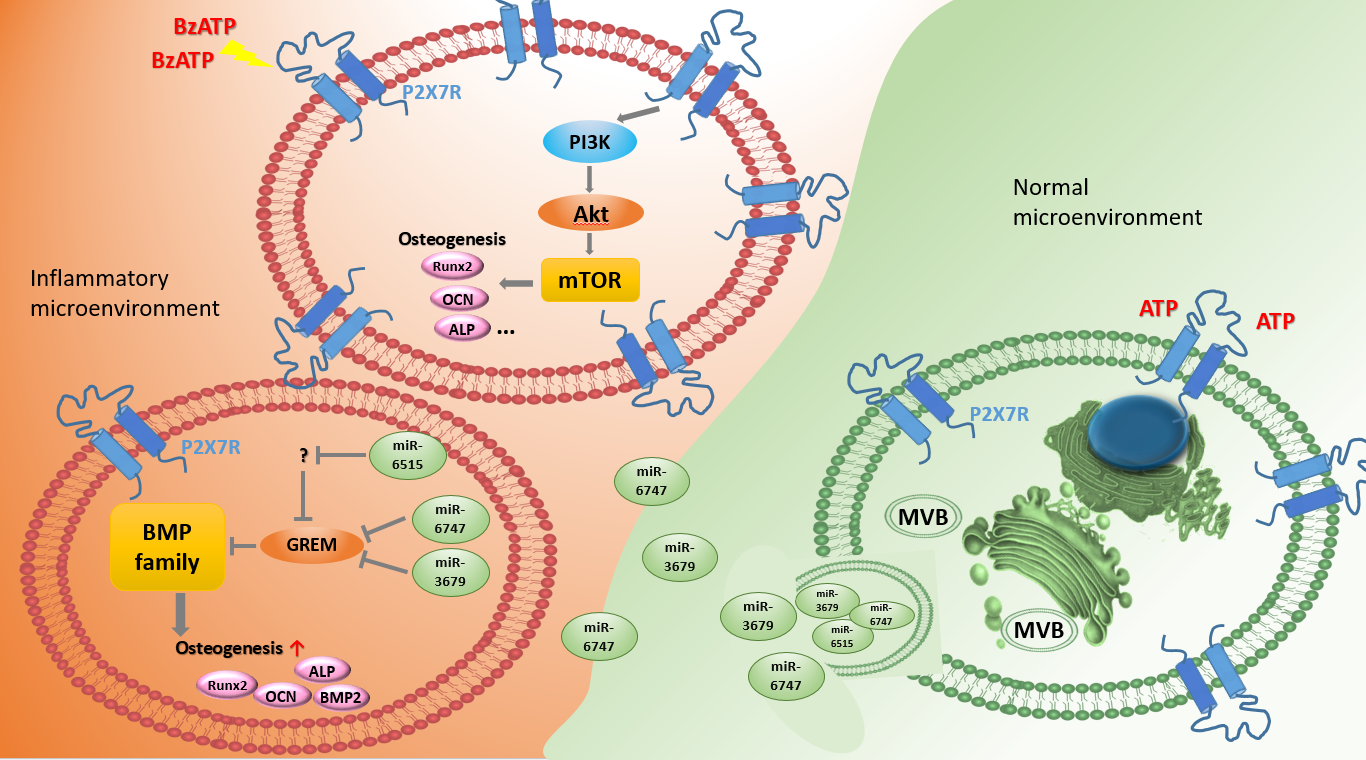


**Supplemental Fig. 4. The potential mechanism involved in P2X7R-mediated rescue of inflammation-compromised PDLSCs from dysfunction.** Schematic illustration of how PI3K-AKT-mTOR signaling, exosomes, miR-3679, miR-6515 and miR-6747 are involved in P2X7R-mediated functional enhancement of PDLSCs living within an inflammatory microenvironment.
